# Functional phosphoproteomic profiling of phosphorylation sites in membrane fractions of salt-stressed *Arabidopsis thaliana*

**DOI:** 10.1186/1477-5956-7-42

**Published:** 2009-11-10

**Authors:** Jue-Liang Hsu, Lan-Yu Wang, Shu-Ying Wang, Ching-Huang Lin, Kuo-Chieh Ho, Fong-Ku Shi, Ing-Feng Chang

**Affiliations:** 1 Graduate Institute of Biotechnology, National Pingtung University of Science and Technology, Pingtung, Taiwan; 2 Mass Solutions Technology Co. Ltd., Taipei, Taiwan; 3 Institute of Plant Biology, National Taiwan University, Taipei, Taiwan; 4 Department of Biological Science, National Taiwan University, Taipei, Taiwan

## Abstract

**Background:**

Under conditions of salt stress, plants respond by initiating phosphorylation cascades. Many key phosphorylation events occur at the membrane. However, to date only limited sites have been identified that are phosphorylated in response to salt stress in plants.

**Results:**

Membrane fractions from three-day and 200 mM salt-treated Arabidopsis suspension plants were isolated, followed by protease shaving and enrichment using Zirconium ion-charged magnetic beads, and tandem mass spectrometry analyses. From this isolation, 18 phosphorylation sites from 15 *Arabidopsis *proteins were identified. A unique phosphorylation site in 14-3-3-interacting protein AHA1 was predominately identified in 200 mM salt-treated plants. We also identified some phosphorylation sites in aquaporins. A doubly phosphorylated peptide of PIP2;1 as well as a phosphopeptide containing a single phosphorylation site (Ser-283) and a phosphopeptide containing another site (Ser-286) of aquaporin PIP2;4 were identified respectively. These two sites appeared to be novel of which were not reported before. In addition, quantitative analyses of protein phosphorylation with either label-free or stable-isotope labeling were also employed in this study. The results indicated that level of phosphopeptides on five membrane proteins such as AHA1, STP1, Patellin-2, probable inactive receptor kinase (At3g02880), and probable purine permease 18 showed at least two-fold increase in comparison to control in response to 200 mM salt-stress.

**Conclusion:**

In this study, we successfully identified novel salt stress-responsive protein phosphorylation sites from membrane isolates of abiotic-stressed plants by membrane shaving followed by Zr^4+^-IMAC enrichment. The identified phosphorylation sites can be important in the salt stress response in plants.

## Background

Under conditions of salt stress, plants respond by activating phosphorylation cascades. For example, the salt overly sensitive (SOS) signaling pathway is known to be involved in stress tolerance in plants [1]. Microarray has long been utilized in transcriptome-based studies to identify salt-induced genes in plants [2-6]. In addition, mass spectrometry-based proteomic studies have also identified salt-induced proteins in plants [7-11]. Both tools provide efficient ways to identify genes or proteins responsive to salt stress. However, the unique protein phosphorylation sites required for the plant response to salt stress have not been well characterized.

Mass spectrometry (MS) is widely used to identify protein phosphorylation sites [12]. Although mass spectrometry coupled with database mining has become a commonly used tool for protein identification, its application to analysis of protein phosphorylation site identification is still far from routine work. Characterization of phosphorylation sites has proven difficult due to both the low abundance of phosphoproteins in living organisms and the suppression phenomenon of phosphopeptides that occurs during MS analysis. Enrichment of phosphopeptides that are low in abundance can circumvent the signal suppression effect caused by nonphosphorylated peptides, allowing for enhanced detection of phosphopeptides by MS. Immobilized metal ion affinity chromatography (IMAC) is a common separation platform used prior to MS analysis for large scale identification of protein phosphorylation sites from complex samples [13]. Typically, phosphopeptides are bound by immobilized metal ions through metal-phosphate affinity interactions, and nonphosphorylated peptides are removed by washing. The phosphopeptides can then be released from the solid support by phosphate or alkaline elution.

Several metal ions have been employed for IMAC, and each metal ion has distinct strengths and weaknesses [14-17]. Among these metal ions, Fe^3+ ^is the most common metal ion used in the IMAC approach; however, its specificity is insufficient for comprehensive phosphoproteome analysis. Recently, Feng *et al*. utilized immobilized zirconium ion affinity chromatography on a polymer-based support to enrich phosphopeptides from mouse liver [18]. According to their previous studies, Zr^4+ ^exhibits higher specificity toward phosphopeptides than Fe^3+ ^[19]. Furthermore, Li *et al*. successfully utilized nitrilotriacetic acid (NTA)-coated magnetic nanoparticles to immobilize Zr^4+ ^for enrichment of histidine-tagged proteins and phosphorylated peptides [20]. In this study, Zr^4+^-IMAC magnetic beads were used to enrich abundant phosphopeptides from milk digest.

Through the use of a metal ion affinity column, phosphorylated peptides can be enriched for phosphorylation site mapping at the proteome level [21]. For example, this strategy was utilized to identify unique phosphorylation sites from elicitor-treated *Arabidopsis *plants [22]. Many groups have also utilized membrane shaving followed by metal ion affinity chromatography and MS analysis to identify phosphorylation sites [15,23-26]. In 2008, the Santoni group utilized MS and identified novel phosphorylation sites in *Arabidopsis *aquaporin PIP2;1 following salt stress [27]. However, the phosphorylation sites of other membrane proteins under the abiotic stress condition are still not well characterized.

To explore the application of Zr^4+^-IMAC magnetic beads to phosphopeptide isolation in a complex sample, we used Zr^4+^-IMAC magnetic beads to enrich phosphopeptides from the membrane fraction of 3-d salt-stressed *Arabidopsis*. To isolate membrane phosphopeptides, we utilized membrane shaving followed by phosphopeptide enrichment. We identified 18 phosphorylation sites, nine of which were from membrane proteins. Label-free and labeling quantitative analyses provided quantitative evidences to support our identification of at least two-fold differential-expressed phosphorylated peptides of membrane proteins. These data suggest that these phosphorylation sites may be important in the salt-stress response in plants.

## Results and discussion

### Mapping of phosphorylation sites from membrane fractions of Arabidopsis through membrane shaving and Zr^4+^-IMAC bead enrichment

In order to identify phosphorylation sites in a model organism, *Arabidopsis thaliana *was chosen as a source of plant material. After crude isolation of total proteins, a further purification was performed using an ultracentrifuge to pellet cellular membranes. Due to their hydrophobicity, membrane proteins are difficult to solubilize for further sample preparation and analysis. Solubilization of membrane proteins using surfactants followed by enzymatic or chemical fragmentation prior to LC-MS/MS analysis is a common approach in the identification of membrane proteins. However, the use of detergents often interferes with chromatographic separation and/or electrospray ionization [28]. According to previous studies, 60% methanol can both promote the solubilization and enhance the proteolysis of membrane proteins [29].

To avoid surfactant interference with phosphopeptide enrichment on Zr^4+^-IMAC beads as well as with liquid chromatography separation, we utilized the organic solvent-assisted approach to shave membranes, followed subsequently by trypsin digestion in mixed organic-aqueous solvent systems. The resulting tryptic digest was then acidified, and the phosphopeptides purified using Zr^4+^-IMAC magnetic beads. Phosphopeptides isolated from membranes of *Arabidopsis *plants treated with varying salt concentrations (0 and 200 mM) were enriched individually within five min of treatment. The IMAC elution fractions from control and salt-treated samples were further analyzed by LC-MS/MS. A summary of all phosphopeptides identified is listed in the Additional file 1.

Overall, 18 phosphorylation sites were identified from 15 proteins in either control (0 mM salt) or salt-treated sample (200 mM salt) in three independent biological replicates R1, R2, and R3 (Table 1, Additional file 2). Table 1 shows sites differentially phosphorylated to salt treatment. Mascot score 38 was used as a cut-off for phosphopeptide identification. To further confirm the identified result, a decoy database search was performed and the FDR was determined to be 0 using Mascot software (the search result were shown in Additional file 1). Salt-treated and salt-free sample were treated and analyzed in parallel. Three independent biological experiments were carried out from sample treatment, sample preparation, and sample analysis. The result showed that some phosphopeptides were identified in each individual experiment but some were identified twice or only once.

**Table 1:** Phosphorylated peptides identified from membrane fractions shaved by protease.

Protein name	GO annotation	ATG number	TMD	Label-free ratio (spectrum)	Label-free ratio (chromatogram)	D/H ratio (spectrum)	D/H ratio (chromatogram)	Phosphopeptide sequence and phosphorylation site
**Membrane protein**								
ATPase 1, plasma membrane-type	ATPase	At2g18960	10	5.62 + 3.01	6.92 + 3.83			GLDIDTAGHHYpTV
Sugar transport protein 1	Transporter	At1g11260		4.36	7.95			GVDDVpSQEF DDLVAASK
Patellin-2	Transporter	At1g22530		2.91 + 0.24	8.37 + 4.03			EILQSEpSFKEEGY LASELQEAEK
Aquaporin PIP2;1, PIP2;2; PIP2;3	Water channel	At3g53420	6	0.33 + 0.10	0.35 + 0.13	0.46	0.49	SLGpSFRpSAANV
		At2g37170						
		At2g37180						
Probable aquaporin PIP2;4	Water channel	At5g60660	6	1.22 + 0.05	1.24 + 0.55			ALGSFGpSFGSFR
Probable aquaporin PIP2;4	Water channel	At5g60660	6	1.22 + 0.05	1.24 + 0.55			ALGSFGSFGpSFR
Probable aquaporin PIP2;4	Water channel	At5g60660	6	3.37 + 1.22	2.38 + 0.15	0.52	0.61	ALGSFGpSFGpSFR
Probable inactive receptor kinase	kinase	At3g02880	2	2.07 + 0.31	3.99 + 1.94	1.68	1.61	LIEEVSHSS GSPNPVpSD
Probable purine permease 18	Transporter	At1g57990	10	7.84 + 0.47	5.31 + 1.03			QTTAEGSANP EPDQILpSPR
**Non-membrane protein**								
Mitogen-activated protein kinase kinase 2	Kinase	At4g29810		1.08	0.61			IISQLEPEVLpSPIKPA DDQLSLSDLDMVK
Uncharacterized TPR repeat-containing protein	Unknown	At1g05150		2.82	4.28			DNDVPVpSY SGSGGPTK
FAM10 family protein	Binding	At4g22670		0.45 + 0.37	0.63 + 0.36	0.54	0.56	VEEEEEEDEIVEpSDVEL EGDTVEPDNDPPQK
FAM10 family protein	Binding	At4g22670		0.20	0.17			SFVVEEpSDDD MDETEEVKPK
60S ribosomal protein L13-1	Translation	At3g49010		1.61 + 0.53	1.28 + 0.41			AGDSpTPEELANA TQVQGDYLPIVR
60S acidic ribosomal protein P1-1, P1-2, P1-3	Translation	At1g01100		0.14 + 0.02	0.24 + 0.02	0.29	0.11	KKDEPAEEpS DGDLGFGLFD
		At5g47700						
		At4g00810						
60S acidic ribosomal protein P2-1, P2-2, P2-4	Translation	At3g44590		0.26 + 0.05	0.24 + 0.02			KEEKEEpSD DDMGFSLFE
		At2g27710						
		At2g27720						
60S acidic ribosomal protein P3-1, P3-2	Translation	At5g57290		0.44 + 0.14	0.68 + 0.27	0.29	0.37	KEEpSEEEE GDFGFDLFG
		At4g25890						
60S acidic ribosomal protein P0-3	Translation	At3g11250		0.12 + 0.04	0.13 + 0.05	0.21	0.27	VEEKKEEpSDEED YEGGFGLFDEE

1. AGI code: the accession number refers to Arabidopsis Gene Idenfier code.

2. TMD: predicted transmembrane domain number.

3. Label-free ratio represents fold difference of peptide level between 200 mM-sample versus control.

4. FDR is calculated 0 based on decoy database search.

5. D/H ratio represents fold difference of peptide level between 200 mM-sample versus control. D: 200 mM; H: control.

6. Each peptide was corroborated by Scaffold using 95% confidence interval as the cut-off.

According to the MS-based analysis, we identified phosphorylation sites from three membrane proteins in both salt-free samples (control) and salt-stressed samples. These membrane proteins, PIP2;2, PUP18 and Y3288, were identified in three independent experiments and regarded as highly abundant phospho-membrane proteins in *Arabidopsis*. Their MS/MS spectra are shown in Additional file 3. In Additional file 3, the doubly phosphorylated peptide, SLGpSFRpSAANV (derived from PIP22_ARATH), was confirmed by the observation of y_n_-196, y_n_-98 and b_n_-98 ions. In Additional file 3, a mixture of two phosphopeptides was observed in MS/MS spectra of samples with different salt treatments. The detection of y_4_'-98 and y_4 _ions in Additional file 3 indicates that the specific phosphorylation sites of both phosphopeptides (LIEEVSHSSGpSPNPVSD and LIEEVSHSSGSPNPVpSD) were not unambiguously resolved by the LC separation.

### Identification of phosphorylation sites in salt-stressed plants

**Figure 1:**
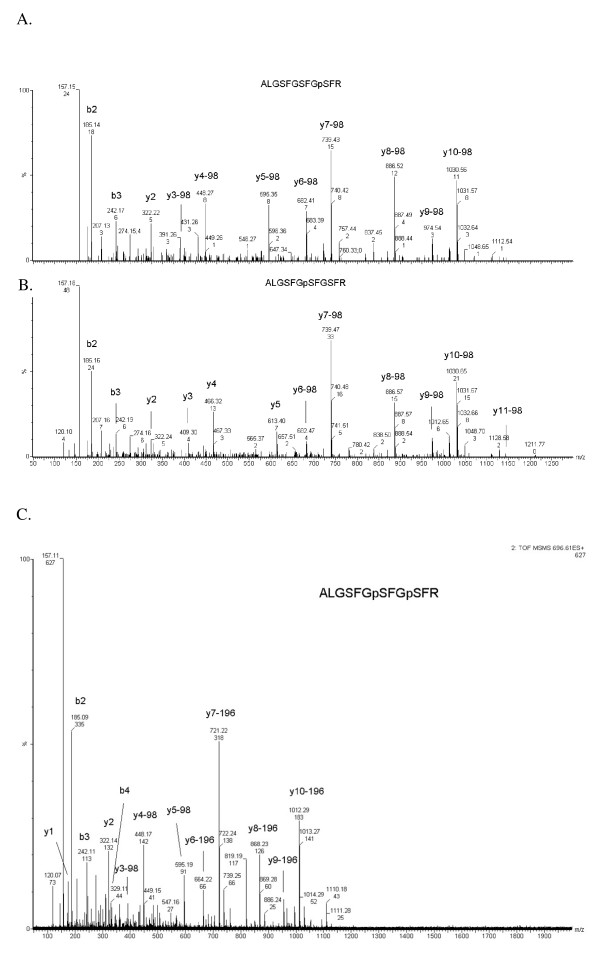
**MS/MS profile of phosphorylated peptides of PIP2;4**. A, MS/MS spectra of PIP2;4 singly phosphorylation site (ALGSFGpSFGSFR); B, MS/MS spectra of PIP2;4 singly phosphorylation site (ALGSFSFGpSFR); C, MS/MS spectra of PIP2;4 doubly phosphorylation sites (ALGSFGpSFGpSFR).

In this study, phosphorylation sites from the membrane protein AtPIP2;4 were observed in either salt-treated or salt-free conditions (Table 1, Additional file 2). In Figure 1A, a phosphorylated peptide from AtPIP2;4 was identified as ALGSFGpSFGSFR due to the observed y_6_-98 and y_5 _ions, which indicated the phosphorylation site was on Ser-283. Another co-eluted phosphorylated peptide from AtPIP2;4 was identified as ALGSFGSFGpSFR due to the clear assignment of y_3_-98 and y_2 _ions shown in Figure 1B, which indicated the phosphorylation site was on Ser-286.. In addition, a doubly phosphorylated peptide ALGSFGpSFGpSFR was identified (Table 1, Figure 1C).

**Figure 2:**
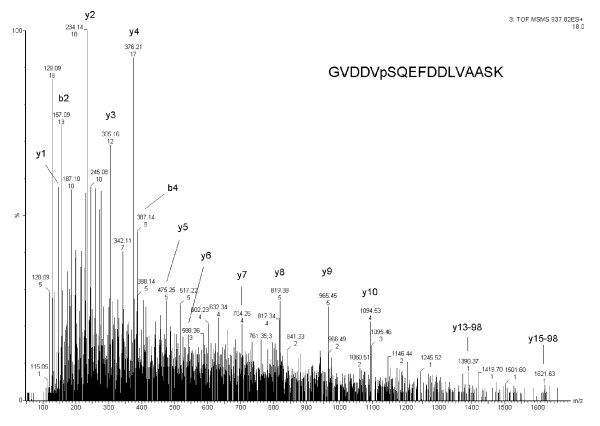
**MS/MS profile of phosphorylated peptide of sugar transporter 1**. MS/MS spectrum of sugar transporter 1 phosphorylation site (GVDDVpSQEFDDLVAASK).

**Figure 3:**
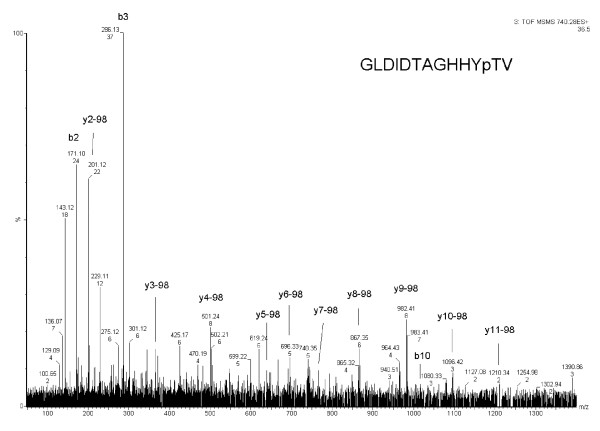
**MS/MS profile of phosphorylated peptide of H^+^-ATPase 1**. MS/MS spectrum of H^+^-ATPase 1 phosphorylation site (GLDIDTAGHHYpTV).

In addition to phosphorylation sites in AtPIP2;4, we also identified a phosphorylation site in membrane protein AtSTP1 (GVDDVpSQEFDDLVAASK) (Table 1, Figure 2) predominately in 200 salt-treated plants. A well characterized phosphorylation site from the C-terminus of membrane protein AHA1 (GLDIDTAGHHYpTV-COOH) was also predominately identified in 200 mM salt-treated Arabidopsis plants (Table 1, Figure 3).

The MS/MS spectra of PATL2, PMA1, and Y1515 corresponding phosphopeptides are shown in Additional file 3, respectively. All phosphorylation sites were confirmed by manual interpretation. These sites were further compared with sites reported in the database, and all were well documented.

#### Quantitative analysis of phosphopeptides enriched by the IMAC magnetic beads

In order to quantify the phosphorylation of membrane proteins under salt-stressed conditions, label-free quantitative analyses were carried out. The label-free quantitative analysis was performed as described in Materials and methods section. All ratios obtained on either MS chromatograms or on MS spectra were summarized in Additional file 4, the peak areas and signal counts were summarized in Additional file 5, and their raw data were also shown in Additional file 1. Among these quantitative data, some phosphopeptides bearing large standard errors may be caused by inconsistent efficiency of phosphopeptide enrichment between individual experiments. When the quantitation data with large standard errors and the data of peptides identified less than twice were neglected, some trends can still be observed. First, all phosphorylated peptides of ribosomal proteins of salt-stressed plants showed reduced level compared to control. However, the reduced level of ribosomal proteins phosphorylation may not conclude to any biological significance due to the possible discordant adsorption toward membrane proteins. Second, most of the identified phosphorylated peptides of membrane proteins showed differential level in responsive to salt stress (Table 1, Additional file 2), such as Patellin-2 and probable purine permease 18.

In addition to label-free quantitative analysis, stable-isotope labeling was applied in this study [29]. The simple and efficient dimethyl labeling was incorporated in the peptide level after the membrane proteins were digested by trypsin. Peptides derived from Control sample was labeled with H form and peptides derived from 200 mM salt-treated sample were labeled with D form then the two pools of sample were combined and purified with Zr^4+^-IMAC magnetic beads. The enriched stable-isotope labeled phosphpeptides were further identified and quantified through LC-MS/MS experiment. This method may eliminate possible system error caused by inconsistent IMAC efficiency in label-free analysis, however, the identified phosphopeptides shown few overlapping to the identified phosphopeptides by label-free approach. This may be due to the reason that stable-isotope labeled peptides shown different chromatographic properties from unlabeled peptides on IMAC and reverse phase (RP) chromatography. Indeed, phosphopeptides which have not been identified label-free approach were identified in this stable-isotope labeling approach, as shown in Additional file 6. In addition, the labeling approach pooled salt-treated sample and salt-free sample together, the overall peptides including common and uncommon peptides will be supposedly more complicated than the individual label-free one, therefore, the enrichment of labeled phosphopeptides on IMAC beads and the separation of labeled phosphopeptides on RP column were supposedly less efficient compared those in label-free analysis. The ratios of either identified phosphopeptides or non-phosphopeptides were summarized in Additional file 6. In Table 1, the quantitation result of labeling approach (D/H ratio) showed similar trends as labele-free approach with an exception of a doubly phosphorylated peptide of PIP2;4 which can be due to the complexity of the multiple phosphorylation sites in a single peptide.

In summary, the quantitative analysis results indicated that the level of phosphopeptides on five membrane proteins such as AHA1, STP1, Patellin-2, probable inactive receptor kinase (At3g02880), and probable purine permease 18 showed at least two-fold increase in comparison to control in response to 200 mM salt-stress.

### Identification of phosphorylation sites by membrane shaving followed by IMAC enrichment

In this study, membrane proteins were efficiently solubilized in the organic solvent-aqueous system allowing trypsin digestion to be performed in the same tube. According to the results acquired using mass spectrometry, the majority of identified proteins were membrane-localized, accompanied by some acidic ribosomal proteins which have been reported to associate with cellular membranes. These results indicated that organic solvent-assisted membrane shaving was appropriate for sample preparation prior to LC-MS/MS analysis. In addition to phosphate groups, sulfonic acid containing-moieties have been reported to be good ligands for the immobilized metal ions used in IMAC approach [30]. By using 60% methanol to solubilize hydrophobic membrane proteins, possible interference from sulfate-containing surfactants was avoided. The use of Zr^4+^-IMAC magnetic beads provided a rapid and efficient platform to enrich phosphopeptides from membrane samples. Using Zr^4+^-IMAC beads, the enrichment process was completed within five minutes, dramatically reducing sample preparation time and allowing for high throughput analysis of protein phosphorylation sites. Therefore, this technique may prove particularly useful for mapping phosphorylation sites in studies exploring time course or dosage effects.

### Identification of a phosphorylation site in the 14-3-3 binding protein AHA1 in 200 mM salt-treated plants

Our study identified a well characterized phosphorylation site in the plasma membrane protein AHA1. The phosphorylated peptide was identified in two of the three biological replicates (Table 1, Figure 3). The label-free quantitative analysis shows an average ratio 6.92 (Table 1), which suggests that this site can be responsive to salt stress. This site is phosphorylated in the C-terminus of many H^+^-ATPases [31]. AHA was found to be a 14-3-3 interacting protein in a tandem affinity purified 14-3-3 complex [32]. The phosphorylated residue (YpTV) can bind 14-3-3 proteins in a phosphorylation-dependent manner [33,34]. The functional importance of this site in pollen has been reported; however, this site has not been characterized in the salt stress response. Although a previous study addresses the importance of the related ATPase AHA4 in salt stress resistance [35], no previous study has provided *in vivo *evidence of the importance of this site in the salt stress response. Our results provide *in vivo *evidence that the phosphorylation site in the C-terminus of AHA1 (GLDIDTAGHHYpTV-COOH) is phosphorylated in 200 mM salt-treated *Arabidopsis *plants. This evidence suggests that phosphorylation of AHA1 plays a role in the salt stress response.

### Identification of a phosphorylation site in a sugar transporter in 200 mM salt-treated plants

Sugar transporters (STP) are plasma membrane proteins that play important roles in the uptake and response to sugar in *Arabidopsis *[36]. In the *Arabidopsis *genome, there are 14 sugar transporter genes. Many of these genes are involved in *Arabidopsis *plant growth and development [37]. For example, the knockout line of the *AtSTP1 *gene exhibits a mutant phenotype characterized by decreased uptake of exogenous monosaccharides by *Arabidopsis *seedlings [36]. The AtSTP1 gene is diurnal and light-regulated and is expressed and localized in guard cells [37]. However, no roles for STP in the salt stress response have been previously reported.

In this study, we identified a phosphorylation site in AtSTP1 (GVDDVpSQEFDDLVAASK) in 200 mM salt-treated plants (Table 1, Figure 2). Nevertheless, this phosphorylated peptide was identified only once in the three biological replicates (Table 1). Since the same site was previously identified in a proteomic study [38], our results confirm the phosphorylation of this residue. The label-free quantitative analysis shows a ratio 7.95 (Table 1), which suggests that this site can be responsive to salt stress. Based on an alignment analysis, this site is not evolutionarily conserved (data not shown). This observation suggests that the function of this phosphorylation site in AtSTP1 in the salt stress response can be unique to *Arabidopsis *and other closely related organisms.

### Identification of phosphorylation sites in aquaporin PIP2;4

The aquaporin water channels are known to be involved in water transport in plant cells [39]. They are encoded by a gene family and can be divided into four subgroups (MIP, TIP, PIP, NIP) [39]. In *Arabidopsis*, the phosphorylation of PIP2;1 has been shown to be regulated by salt [40]. In this study, two phosphorylation sites in PIP2;4 (Ser-283 and Ser-286) were identified in either salt-treated or salt-free samples (Table 1, Figure 1) but the two mono-phosphorylated peptides, ALGSFGpSFGSFR and ALGSFGSFGpSFR were co-eluted during the LC-MS/MS analysis that the resulting data was contributed by the combination of two individual phosphopeptides (Table 1). Therefore, the exact expression ratios of these two individual sites were not clear. These two sites are novel sites and were not previously reported.

The doubly phosphorylated peptide, ALGSFGpSFGpSFR, was also identified in this study. According to label-free analysis, this doubly phosphorylated peptide was identified predominately in salt-treated condition. The ratios of three independent experiments were 2.49, 2.57, and 2.08 respectively. Nevertheless, according to labeling analysis, the ratio was 0.52. This indicated that this doubly phosphorylation was effected by salt stimulation. The inconsistent ratios between two independent analysis methods may be due to the complexity of the multiple phosphorylation sites in a single peptide. Understanding the functional significance of Ser-283 and Ser-286 phosphorylation in the salt-stress response requires further study.

### Identification of a phosphorylation site in a MAPKK in 400 mM salt-treated plants

**Figure 4:**
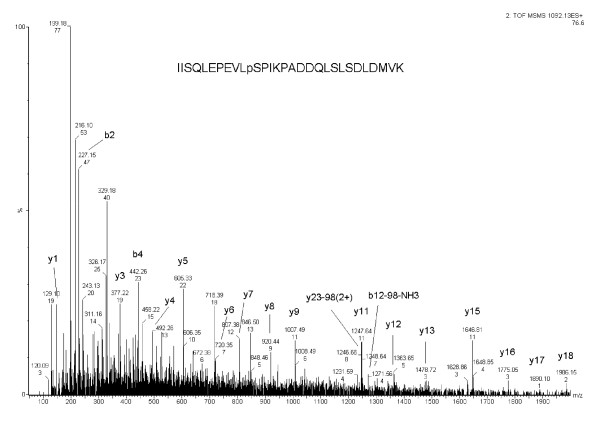
**MS/MS profile of phosphorylated peptide AtMPKK2**. MS/MS spectrum of AtMPKK2 phosphorylation site (IISQLEPEVLpSPIKPADDQLSLSDLDMVK).

The MAPK signaling pathway has been reported to be involved in the osmotic stress response [41]. In *Arabidopsis *a MAPKK, AtMKK2, has been shown to be involved in salt stress signaling [42]. The same kinase was also found to play a role in disease resistance [43]. AtMKK2 is activated by *Arabidopsis *MEKK1 [42]. However, the phosphorylation sites of these kinases have not been well characterized. In this study, we identified a phosphorylation site (IISQLEPEVLpSPIKPADDQLSLSDLDMVK) in the salt-regulated MAPKK, MKK2, predominately in 400 mM salt-treated *Arabidopsis *plants (Figure 4). The label-free ratio is 4.43 in comparison to control (data not shown). Although a putative phosphorylation site in Arabidopsis MKK2 has previously been reported [44], the site we identified is in a different position and has not been previously documented.

Based on an alignment analysis, this site is conserved among different plant species (data not shown). Although the MAPKK (SIMK kinase) of a salt-induced MAPK [45] has also been identified in alfalfa [46], the phosphorylation site is not conserved between the two, suggesting that these two proteins may participate in independent salt signaling pathways. Since this site was identified only in the 400 mM salt-treated plants, this phosphorylation event may be regulated selectively by high salt conditions.

### Identification of a phosphorylation site in a calcium-binding protein

A salt-treatment specific phosphopeptide was identified from a novel *Arabidopsis *protein (At1g05150) containing two conserved motifs: the TPR motif and the EF-hand. This phosphorylated peptide was identified in all three biological replicates (Table 1). The TPR motif is known to be involved in protein-protein interactions [47]. This protein also has two predicted EF-hands, which are generally involved in calcium binding. The phosphorylation site is novel and has not previously been shown. The identification of the phosphorylation site of this protein suggests the possible involvement of both calcium and phosphorylation in regulation of salt stress signaling.

### Phosphorylation sites identified in both control and salt-treated Arabidopsis plants

Phosphorylation sites in aquaporins were recently broadly characterized. These sites are generally found at the C-terminus of the protein [39]. In our study, phosphorylation sites in PIP2;1, PIP2;2 and PIP2;3 were found in both control and salt-treated plants. In addition to aquaporin PIP2, the phosphorylation sites of a receptor-like kinase (At3g02280) and a purine permease (At1g57990) were also identified in both control and salt-treated plants. These phosphorylated peptides were identified in all three biological replicates (Table 1). These phosphorylation sites have previously been described [38,48], consistent with our MS data. Santoni group utilized stable isotope labeling to qnantify phosphorylation site of PIP2;1 and detected reduced phosphorylation level in the salt-stressed Arabidopsis [39]. In our quantitative analyses, we observed a quantitative ratio around 0.3. Our data supported Santoni's results.

### Sequence alignment of C-terminus of aquaporin PIP2 in plants reveals evolutionarily conserved and variable phosphorylation sites

**Figure 5:**
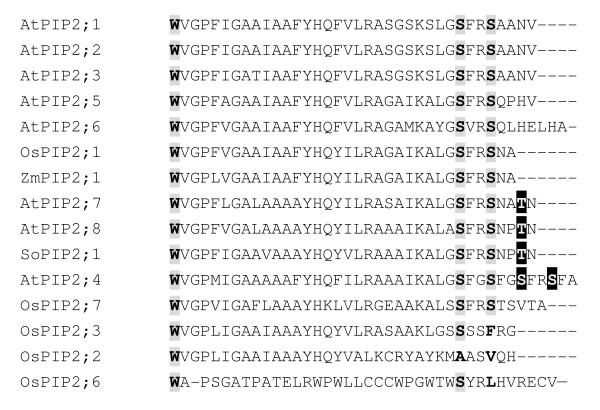
**Alignment of the amino acid sequences of aquaporin from different plants**. Multiple amino acid sequence alignment of aquaporin in *Arabidopsis thaliana *(At), *Oryza sativa *(Os), *Spinacia oleracea *(So) and *Zea mays *(Zm). Highly conserved phosphorylation sites are highlighted in gray; Black area represents conserved phosphorylation sites.

In order to determine whether the phosphorylation site of aquaporin AtPIP2;4 is evolutionarily conserved, we carried out a sequence alignment of the C-terminus of PIP2s from Arabidopsis and rice. In addition, the C-terminus of spinach SoPIP2;1 was also included since its phosphorylation site was reported. Based on the alignment result, a Ser phosphorylation site corresponding to Ser274 in spinach SoPIP2;1 [49] is evolutionarily conserved among all PIP2 proteins (Figure 5). This phosphorylation site was also found to be phosphorylated in maize PIP2;1 [50], rice PIP2s [25], and Arabidopsis PIP2s [40]. Calcium-dependent protein kinase (CDPK) was demonstrated to be the kinase that phosphorylates the site ([51]-[52]).

Based on the sequence alignment, a Ser phosphorylation site corresponding to Ser277 in spinach SoPIP2;1 [49] is evolutionarily conserved among some PIP2 proteins (Figure 5). This site was found to be phosphorylated in spinach SoPIP2;1 [49] and in Arabidopsis AtPIP2;1 [40]. In this study, we identified a phosphorylation site of AtPIP2;4 (Ser283) corresponding to Ser277 in spinach SoPIP2;1 (Figure 5). This site was identified only in the control sample. Our result shows that this site is also a conserved site.

By contrast, in this study we identified another phosphorylation site of aquaporin AtPIP2;4 (Ser286) which was found only in salt-treated samples. Based on the sequence alignment, this site is not conserved among PIP2 family members (Figure 5). Since this phosphorylation site was not published before, we therefore concluded that this site is a novel one. We suspect that the phosphorylation of this novel site can be regulated by either a CDPK or an unknown kinase since this site does not have a consensus CDPK phosphorylation motif.

### Identification of receptor-like kinase At3g02880

**Figure 6:**
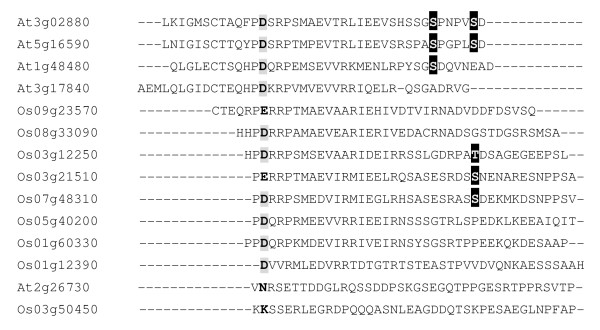
**Alignment of the amino acid sequences of receptor-like kinase At3g02880 *in planta***. Amino acid sequence alignment of C-terminal of membrane proteins in *Arabidopsis thaliana *(At) and *Oryza sativa *(Os). Conserved amino acids are highlighted in gray. Black area represents the phosphorylation sites.

Receptor kinase At3g02880 is a member of receptor-like kinase family. This gene family is involved in signaling in plant cells [53]. Nevertheless, little is known about the functions of the family members. The phosphorylated peptides of receptor kinase At3g02880 were identified in control and salt-stressed samples. The average label-free ratio 3.99 and D/H ratio 1.61 were both higher than 1, which suggests that the phosphorylation sites can be involved in salt-stress signaling. Based on the sequence alignment, the phosphorylation site is conserved among receptor kinase family members in rice and Arabidopsis (Figure 6). This suggests these sites may have important biological functions in plants.

## Conclusion

In this study we show that membrane shaving followed by Zr^4+^-charged magnetic bead phosphopeptide isolation can successfully identify phosphorylation sites in membrane and membrane-associated proteins. In particular, we identified several phosphorylated peptides containing sites which appear to be regulated by salt. These phosphorylation sites may therefore be important in salt-stress responses in plants.

## Materials and methods

### Chemicals

Acetonitrile, acetic acid, ammonium bicarbonate, formaldehyde (37% in H2O), and ammonium hydroxide (28~30%) were purchased from J. T. Baker (Phillipsburg, NJ, USA). 1,4-Dithiothreltol (DTT, Cleland's reagent) and methanol were obtained from Mallinckrodt Baker (NJ, USA). Formic acid and sodium acetate were purchased from Riedel-de Haën (Seelze, Germany). Iodoacetamide (IAA), sodium cyanoborohydride and formaldehyde-d_2 _(20% solution in D_2_O) were purchased from Sigma (MO, USA). Zr^4+^-IMAC magnetic beads were provided by Mass Solutions Technology Co. Ltd (MST, Taipei, Taiwan). Sequencing grade trypsin was purchased from Promega (Madison, WI, USA). The water used in the protein digestion and purification experiments was obtained using the Direct-Q™ pure water purification system (Millipore, MA, USA). Formaldehyde is known to the state of California to cause cancer; special caution was taken including the use of surgical gloves and fume hood when handling formaldehyde.

### Seed sterilization and germination

*Arabidopsis thaliana *(ecotype Columbia) seeds were sterilized in a chamber with Cl_2 _gas which was created by adding 3 ml 12N HCl to the beaker containing 100 ml 12% NaOCl. After 3 hours, the chamber cap was opened for 0.5 hour. After closing the cap of the microfuge tube, the chamber was exposed to laminar flow for 0.5 hour to clear the Cl_2 _gas. Finally, the cap was closed and the sterilized seeds were stored at 4°C. Sterilized seeds were planted on plates containing 0.5 × Murashige and Skoog (MS) medium supplemented with 0.5% sucrose, pH 5.7. After sealing the plates with ventilative adhesive tape, seeds were incubated at 4°C in the dark. One week later, seeds were transferred to the growth chamber (22°C/16-h photoperiod, 100 μE m^-2 ^s^-1 ^light) for germination.

### Seedling suspension culture and salt treatment

**Figure 7:**
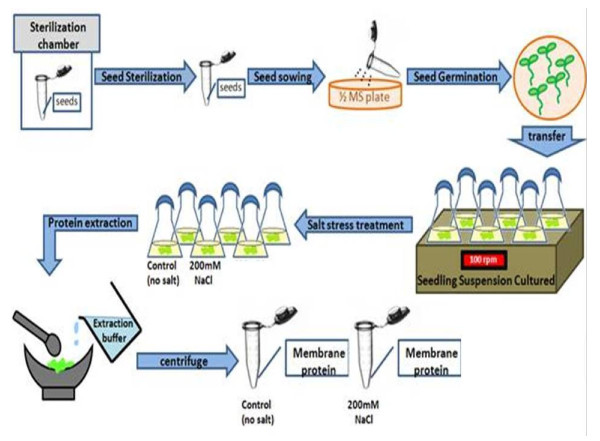
**Suspension culture of Arabidopsis plants and protein purification**. Arabidopsis seedlings were suspension cultured followed by a 3-d salt (0, 200 mM NaCl) treatment. Membrane proteins were harvested by pellet total proteins using ultracentrifugation.

After seed germination, seedlings were transferred to a 500 ml flask containing 200 ml suspension culture media (0.5 × Murashige and Skoog medium supplemented with 0.5% sucrose, pH 5.7). The seedlings were incubated at twilight (25°C/16-h photoperiod, 10 μE m^-2 ^s^-1 ^light) in suspension cultures (100 rpm) to enrich root growth. After 4 weeks, the suspension culture media was renewed by laminar flow. Plants were treated with either no salt (Control) or 200 mM NaCl (Figure 7) for three days.

### Membrane protein purification

After three-day salt treatment, plants were harvested and washed three times with Reverse Osmosis water. After drying the plants, the weights of the plants were recorded. Liquid nitrogen was use to pre-cool the mortar, and plant tissue was ground to a fine powder. Two ml extraction buffer [20 mM Tris, pH 7.5; 100 mM NaCl; 10% glycerol; 1× protease inhibitor cocktail (Complete with EDTA, Roche)] was added to the mortar containing 1 mg of plant tissue in the presence of continued grinding to increase extraction efficiency. The crude extract was filtered using Miracloth at 4°C. The crude extract was then centrifuged (Beckman J2-MC) for 30 min at 4°C to collect the supernatant. The supernatant was centrifuged again in an ultracentrifuge (Beckman L8-M) for 75 min at 4°C to collect the membrane fractions (Figure 7). Concentrations of membrane proteins were measured using the Bradford assay. Three independent biological replicates (R1, R2, and R3) of membrane fractions for control and salt-treated plants were prepared and analyzed.

### Membrane shaving by trypsin digestion

Membrane samples treated with different salt concentrations were resuspended and digested individually using trypsin as previously reported [54]. Briefly, samples (each approximately 200 μg) were resuspended individually in 50 mM ammonium bicarbonate, pH 8.0, via vortexing and sonication. The proteins were thermally denatured at 90°C for 20 min, then cooled to room temperature and diluted with methanol to produce a composition of 60% organic solvent. Sequencing grade trypsin (5 μg) was added to the organic-aqueous buffer, and the mixture was incubated at 37°C overnight. The proteolysis reaction was quenched by rapid freezing and stored at -80°C prior to Zr^4+^-IMAC enrichment.

### Enrichment of phosphopeptides by Zr^4+^-IMAC magnetic beads

The enrichment of phosphopeptides was carried out using Zr^4+^-IMAC beads according to the manufacturer's instructions. Briefly, the tryptic digested membrane fraction was diluted in 100 μL of incubation buffer (200 mM NaCl/10% HOAC/20% ACN) and added to 20 μL of Zr-IMAC bead suspension. The suspension was vigorously mixed by pipetting in and out of a sample vial for 30 sec. The beads were then concentrated using a magnet, and the supernatant was removed. The magnetic beads were washed in 100 μL of incubation buffer (200 mM NaCl/10% HOAC/20% ACN) to remove most nonphosphopeptides. After vigorous mixing by pipetting for 30 sec, the beads were concentrated using a magnet, and the supernatant was removed by pipetting. The wash was repeated twice. The magnetic beads were then rinsed with 20 μL of 1% ammonium hydroxide in 50 mM ammonium bicarbonate to elute bound phosphopeptides. After vigorous mixing for 30 sec, the supernatant was collected by magnetic separation, and the aliquot was further analyzed by LC-MS/MS.

### LC-MS/MS analysis

The resulting peptide mixture was subjected to the CapLC system (Waters, Milford, MA) utilizing a capillary column (75 μm i.d., 10 cm in length, MST, Taiwan) with a linear gradient from 5% to 50% acetonitrile containing 0.1% formic acid over 46 min. The separated peptides were on-line analyzed under positive survey scan mode on a nano-ESI Q-TOF (Micromass, UK) instrument. The scan range was from m/z 400 to 1600 for MS and m/z 50 to 2000 for MS/MS. The raw data was processed into a PKL text file format using MassLynx 4.0 (subtract 30%, smooth 3/2 Savitzky Golay and center three channels in 80% centroid).

### Label-free quantitative analysis

A label-free quantitative analysis was performed using parallel LC-MS/MS runs. The relative ratio was analyzed manually through MassLynx 4.0. Briefly, the precursor ions of the corresponding phosphopeptides were selected by SIC mode and displayed as BPI chromatograms. Each peak of the corresponding m/z on MS chromatogram was correlated with the retention of its precursor on MS/MS chromatogram. The peaks of phosphopeptides derived from salt-treated and salt-free conditions were displayed at a similar retention time in parallel chromatograms. The relative ratio of phosphopeptide with and without salt treatment was determined by comparison with peak areas in different MS chromatograms at an almost the same retention time. Each chromatogram was processed by subtraction and smoothing prior to comparison. Alternatively, the ratio determination also can be achieved on MS spectrum rather than MS chromatogram. The MS spectra of salt-treated and salt-free samples were displayed at the similar retention time interval, and the ratio of the same precursor was determined by intensity comparison of each corresponding monoisotopic peak.

### Quantitative analyses of phosphorylated peptides by dimethyl labeling, IMAC enrichment and LC-MS/MS

Quantitative analyses of phosphorylated peptides by dimethyl labeling were carried out as previously described [55]. The peptide mixture derived from membrane fraction without salt stress was dissolved in 50 μL of sodium acetate buffer (100 mM, pH 5-6) and mixed with formaldehyde (4% in water, 2 μL), then mixed immediately with freshly prepared sodium cyanoborohydride (600 mM, 1 μL). The mixture was vortexed again and then allowed to react for 5 min. If necessary, ammonium hydroxide (4% in water, 2 μL) was added to consume the excess aldehyde. The peptide mixture derived from 200 mM salt stressed membrane fraction was labeled with deuterium form in a similar manner, but by using formaldehyde-d_2 _(4% in water, 2 μL). The H-labeled (control) and D-labeled (200 mM salt-treated group) samples were combined and acidified by 10% acetic acid to around pH 3. The stable-isotope labeled phosphopeptides were enriched by Zr^4+ ^IMAC magnetic beads according the protocol mentioned above. The resulting elute fraction from IMAC enrichment was then analyzed by LC-MS/MS.

### Database and statistical analysis

For protein identification, the PKL files generated from MS/MS spectra were uploaded to the MASCOT search engine v2.2 (Matrix Science, UK) http://www.matrixscience.com[56]. The parameters for database searching were as follows: (1) Protein database was set to be SwissProt; (2) Taxonomy was set as *Arabidopsis thaliana *(thale cress); (3) One trypsin missed cleavage was allowed; (4) The mass tolerance was set to be 0.4 Da for both precursor and product ions; (5) Phospho (ST), Phospho (Y), deamidated (NQ), oxidation (M), Dimethyl (K), Dimethyl (N-term), Dimethyl:2H(4) (K), Dimethyl:2H(4) (N-term) were chosen for variable modifications; (6) Data format was chosen as Micromass (.pkl) and instrument was chosen as ESI-QUAD-TOF. (7) Proteins with scores above the significance threshold (p < 0.05) were shown as identified proteins. All MS/MS spectra of identified phosphopeptides were further verified by manual interpretation. Scaffold software was used to provide validation and confidence level (% probability) of the identification and 95% confidence level was used as the cut-off. The false discovery rate (FDR) was calculated using a decoy database search [57]. The identification of dimethyl labeled peptides were directly performed on MASCOT search engine by choosing Dimethyl (K) and Dimethyl (N-term) indicated H-labeling and choosing Dimethyl:2H(4) (K) and Dimethyl:2H(4) (N-term) indicated D-labeling. The D/H ratio was calculated from the relative intensities of D-labeled and H-labeled peptides on mass spectrum. A label-free quantitative analysis was performed using several LC-MS/MS runs. The relative ratio was determined by comparison with peak intensities in different LC-MS runs under strict retention time alignment.

### Bioinformatic analysis of phosphorylation sites

BLAST searches [58] were performed to identify protein homologues in plants. To determine the localization of phosphorylation sites in a membrane protein, results were used to query the ARAMEMNON database http://aramemnon.botanik.uni-koeln.de/index.ep[59]. This step was used to confirm that the identified sites were not located within transmembrane domains. To discover if phosphorylation sites had been previously identified, sites were compared to results from PhosphoPhat http://phosphat.mpimp-golm.mpg.de/, P3DB http://www.p3db.org/ and PepBase http://pepbase.iab.keio.ac.jp/phospho/msb/[60-62].

Protein sequence alignment was performed using ClustalW software to align phosphoprylation sites http://align.genome.jp/. Amino acid sequences of Arabidopsis aquaporin, AtPIP2;1 (At3g53420), AtPIP2;2 (At2g37170), AtPIP2;3 (At2g37180), AtPIP2;4 (At5g60660), AtPIP2;5 (At3g54820), AtPIP2;6 (At2g39010), AtPIP2;7 (At4g35100) and AtPIP2;8 (At2g16850), and rice aquaporin, OsPIP2;1 (Os07g26690), OsPIP2;2 (Os02g41860), OsPIP2;3 (Os04g44060), OsPIP2;6 (Os04g16450) and OsPIP2;7 (Os09g36930), were retrieved from TAIR database http://www.arabidopsis.org/index.jsp and Rice Genome Annotation database http://rice.plantbiology.msu.edu/, and spinach and maize aquaporin sequences were blasted against NCBI database http://www.ncbi.nlm.nih.gov/. According to the identity of amino acid sequence of At3g02880, other amino acid sequences of Arabidopsis membrane proteins, At5g16590, At1g48480, At3g17840, At2g26730, and rice membrane proteins, Os03g12250, Os03g21510, Os09g23570, Os07g48310, Os05g40200, Os01g60330, Os08g33090, Os01g12390, Os03g50450, were blasted using TAIR database http://www.arabidopsis.org/index.jsp and Rice Genome Annotation database http://rice.plantbiology.msu.edu/. Amino acid sequence alignment was retrieved from ClustalW http://align.genome.jp/.

## Competing interests

The authors declare that they have no competing interests.

## Authors' contributions

JLH established the analytical workflow for the identification of protein phosphorylation, including the digestion of membrane proteins, the enrichment of phosphopeptides, the LC-MS/MS analysis of phosphopeptide and the downstream Mascot database search. In addition, the manual validation of MS/MS spectra for all phosphopeptides was also achieved by this author. LYW did peptide sample preparation prior to LC-MS/MS analysis, IMAC magnetic beads preparation, phosphopeptide purification, and most LC-MS/MS experiments involved in this work. SYW did seed germination, liquid culture, and salt treatment for Arabidopsis plants. She purified membrane proteins by use of ultracentrifugation followed by membrane shaving. KCH provided chemicals for the liquid culture. FKS provided solutions for the problems encountered in LC-MS/MS experiments in this study. IFC participated in the overall idea and experimental design, QC of data analysis, coordination and preparation of the final version of the manuscript. All authors read and approved the final manuscript. This manuscript has been proofread and edited by native English speakers with related background in BioMed Proofreading.

## Supplementary Material

Additional file 1 **Raw data of Peak area and MS intensity**. The file includes peak area and MS intensity of each identified phosphopeptide. Click here for file

Additional file 2 **Phosphorylated peptides identified from membrane fractions shaved by protease**. This file includes detailed quantitative amounts for each identified phosphopeptide in each biological replicate. Click here for file

Additional file 3 **MS/MS spectrum of six phosphopeptides**. The file includes MS/MS spectrum of SLGpSFRpSAANV (PIP22_ARATH); MS/MS spectrum of QTTAEGSANPEPDQILpSPR (PUP18_ARATH); MS/MS spectrum of LIEEVSHSSGSPNPVpSD and LIEEVSHSSGpSPNPVSD mixture (Y3288_ARATH); MS/MS spectrum of EILQSEpSFKEEGYLASELQEAEK (PATL2_ARATH); MS/MS spectrum of GLDIDTAGHHYpTV (PMA1_ARATH); MS/MS spectrum of DNDVPVpSYSGSGGPTK (Y1515_ARATH). Click here for file

Additional file 4 **Summary of ratios**. This file includes summary of ratios. Click here for file

Additional file 5 **Summary of peak intensity**. This file includes summary of peak intensity. Click here for file

Additional file 6 **Quantitative analysis by labeling**. This file includes summary of ratios by the labeling method. Click here for file
