# Supplementary material for: Functional phosphoproteomic profiling of phosphorylation sites in membrane fractions of salt-stressed Arabidopsis thaliana
Source: Proteome Sci. 2009 Nov 10;7:42. doi: 10.1186/1477-5956-7-42 (PMC2778640; doi:10.1186/1477-5956-7-42)
Supplement: Additional file 1 — Raw data of Peak area and MS intensity. The file includes peak area and MS intensity of each identified phosphopeptide. [file 1477-5956-7-42-S1.doc]

Peak area and MS intensity raw data of Aquaporin PiP2;1

1. SLGpSFRSAANV (1^st^ run –SIC chromatogram and MS spectra)


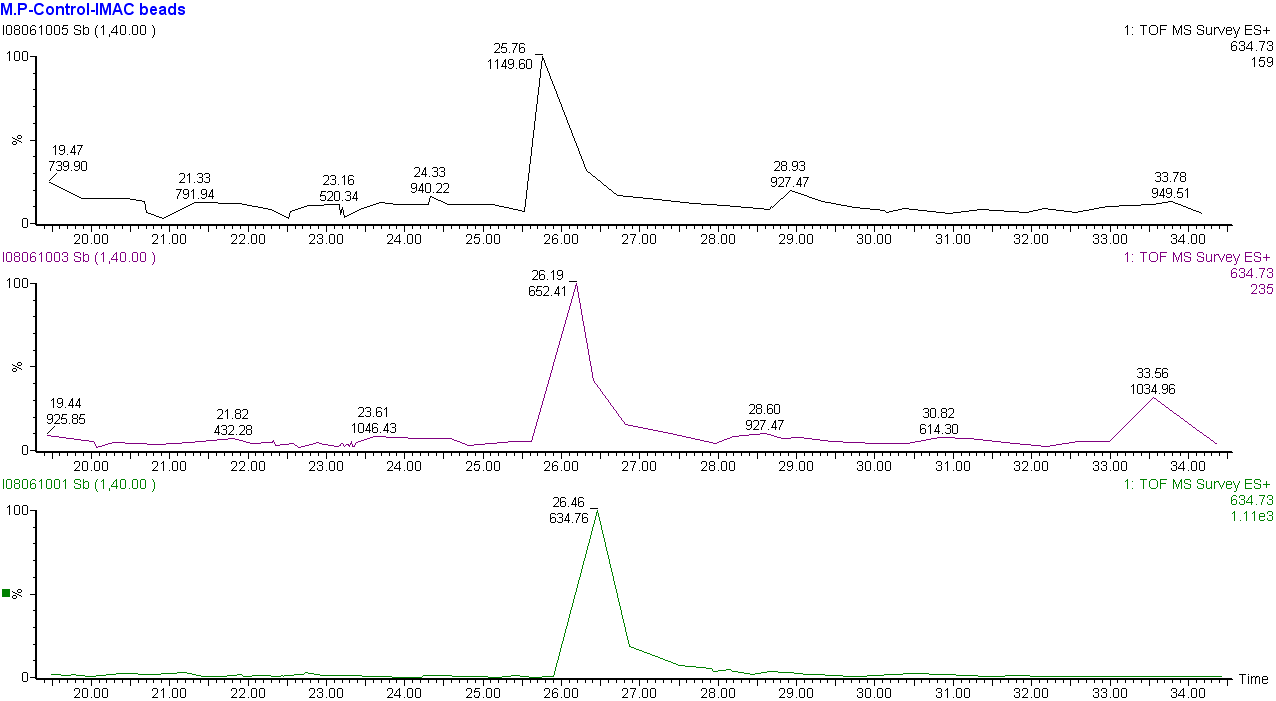


Control

200mM

400mM


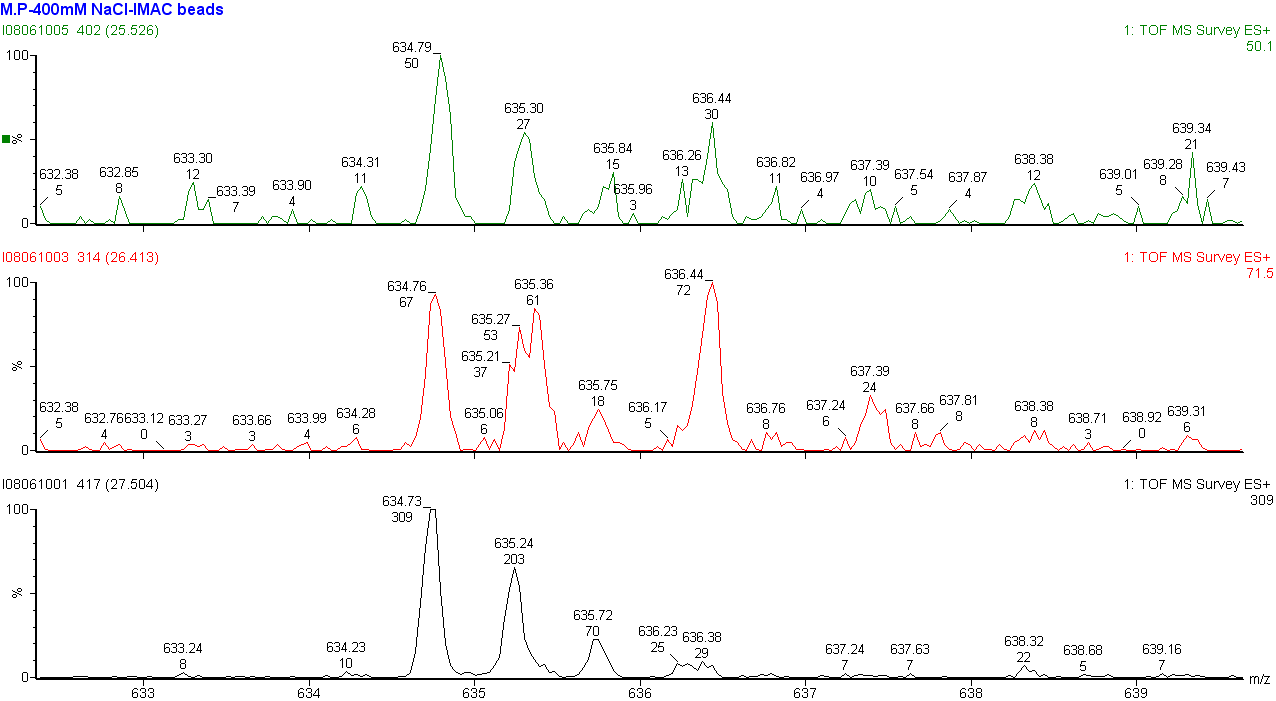


400mM

200mM

control

SLGpSFRSAANV (2^nd^ run –SIC chromatogram and MS spectra)


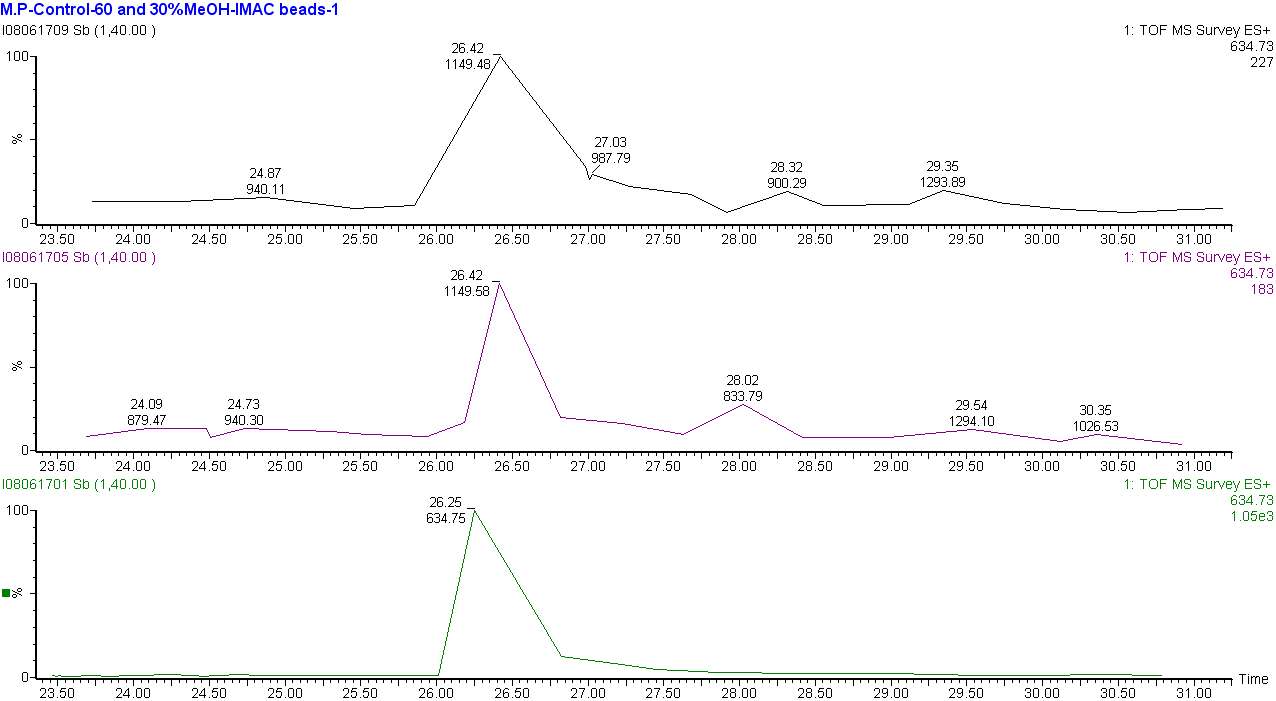


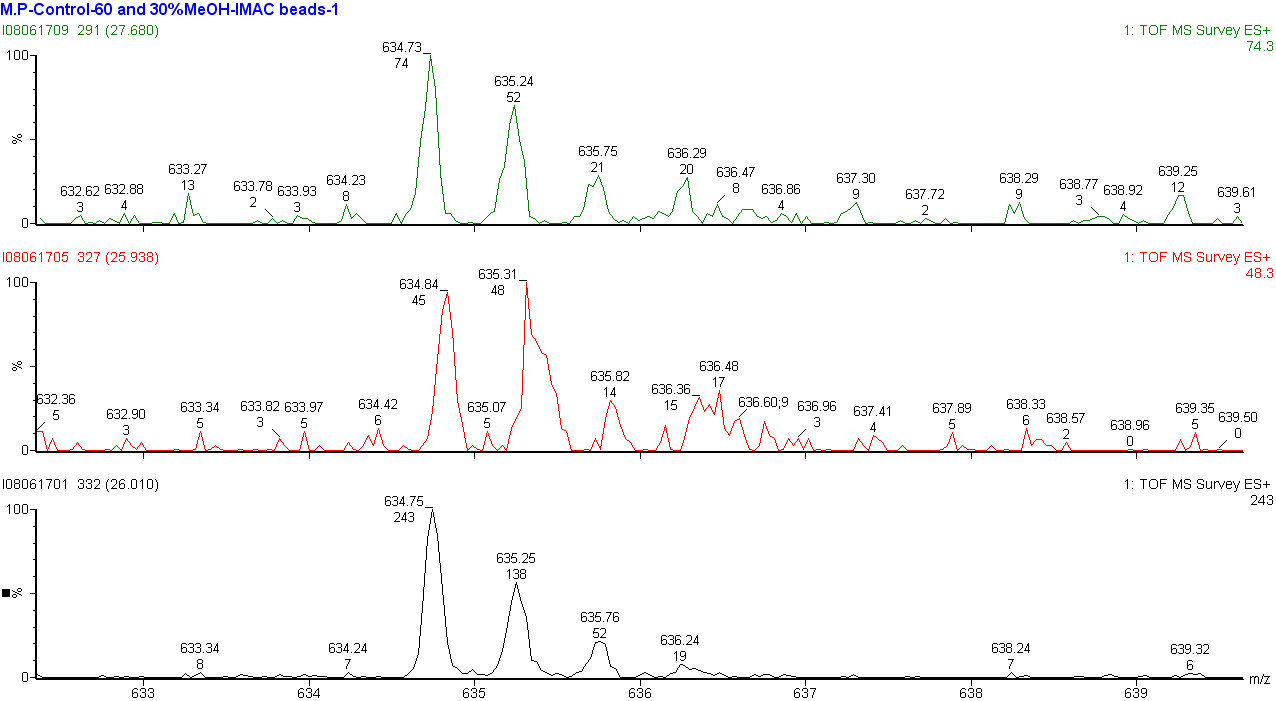


SLGpSFRSAANV (3^rd^ run –SIC chromatogram and MS spectra)


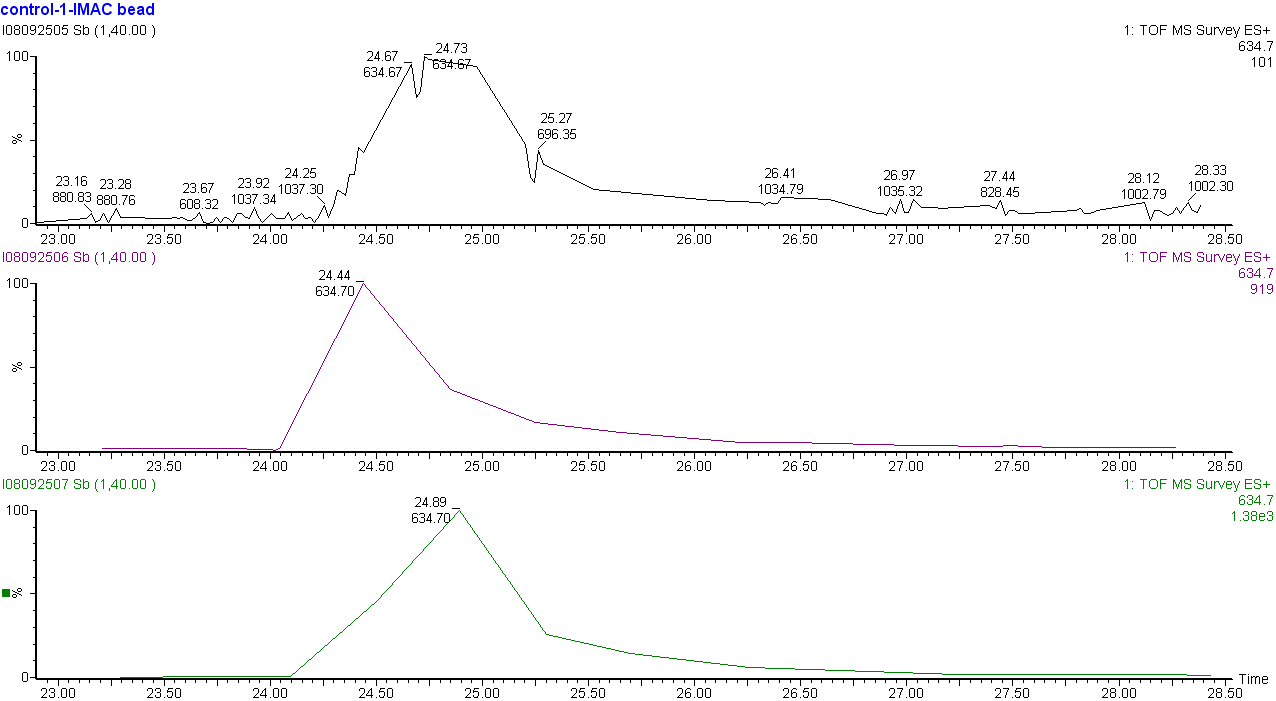


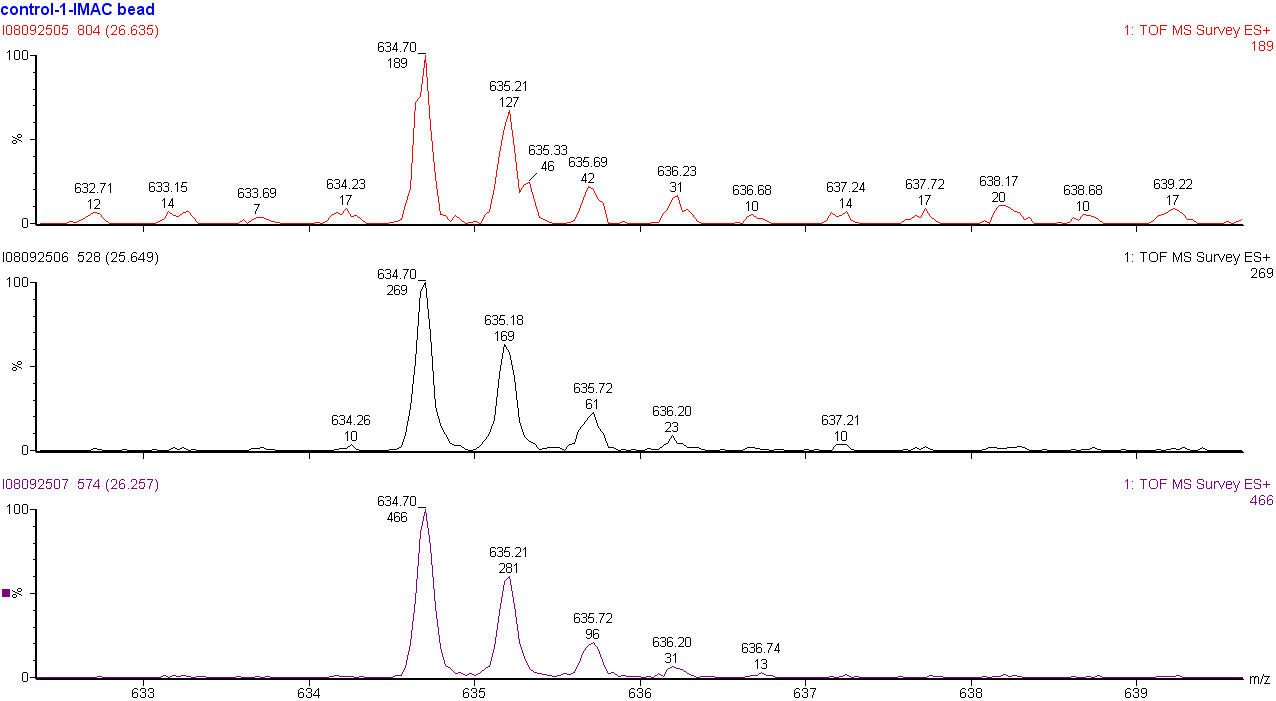


Peak area and MS intensity raw data of ATPase 1

GLDIDTAGHHYpTV (1^st^ run –SIC chromatogram and MS spectra)


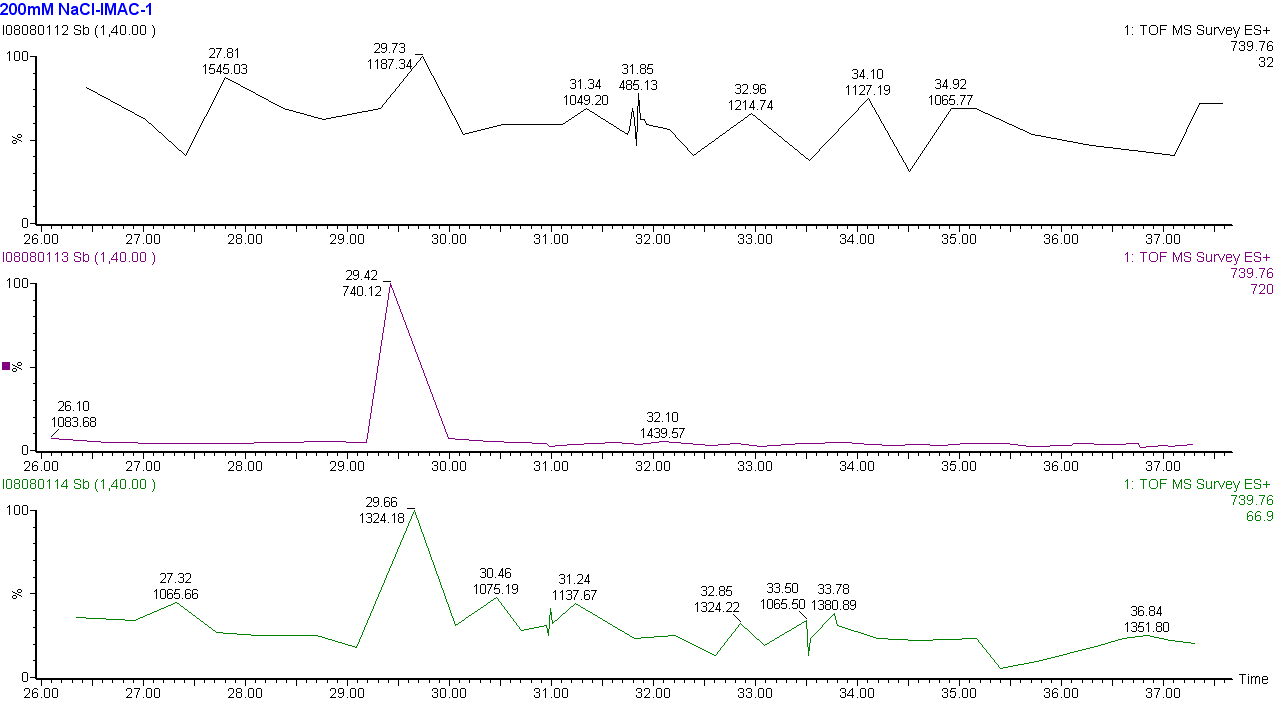


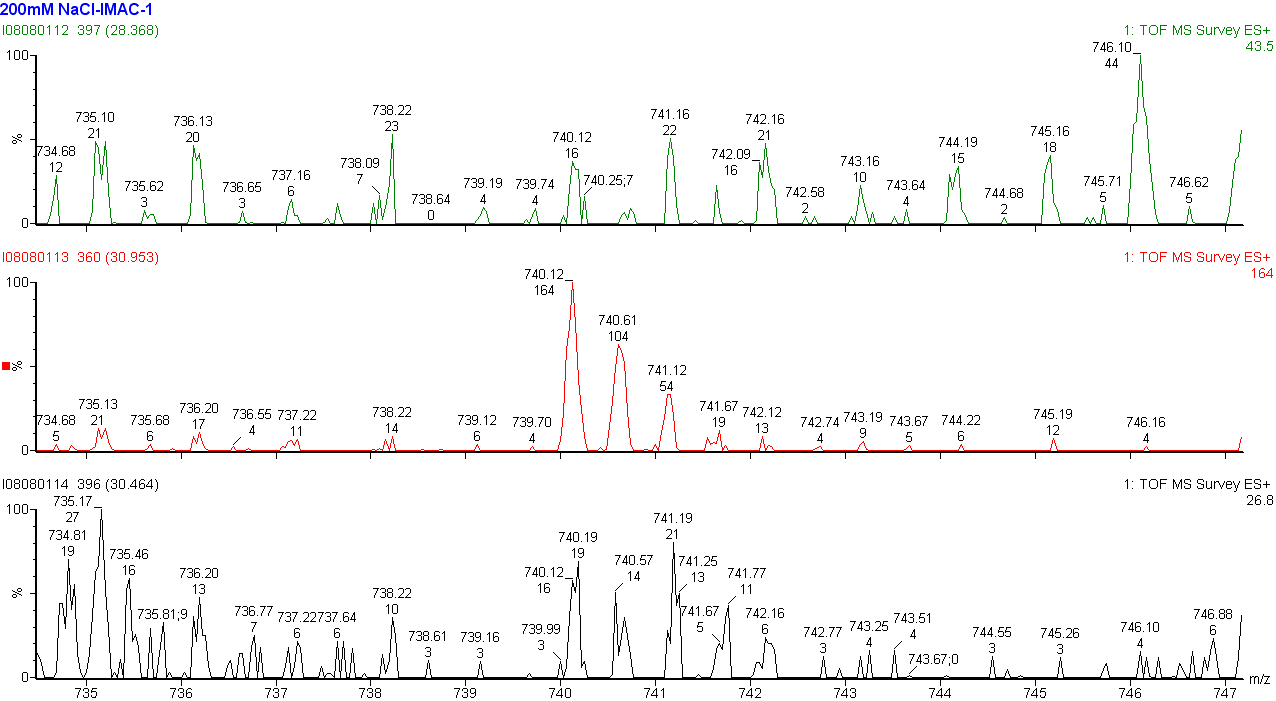


GLDIDTAGHHYpTV (2^nd^ run –SIC chromatogram and MS spectra)


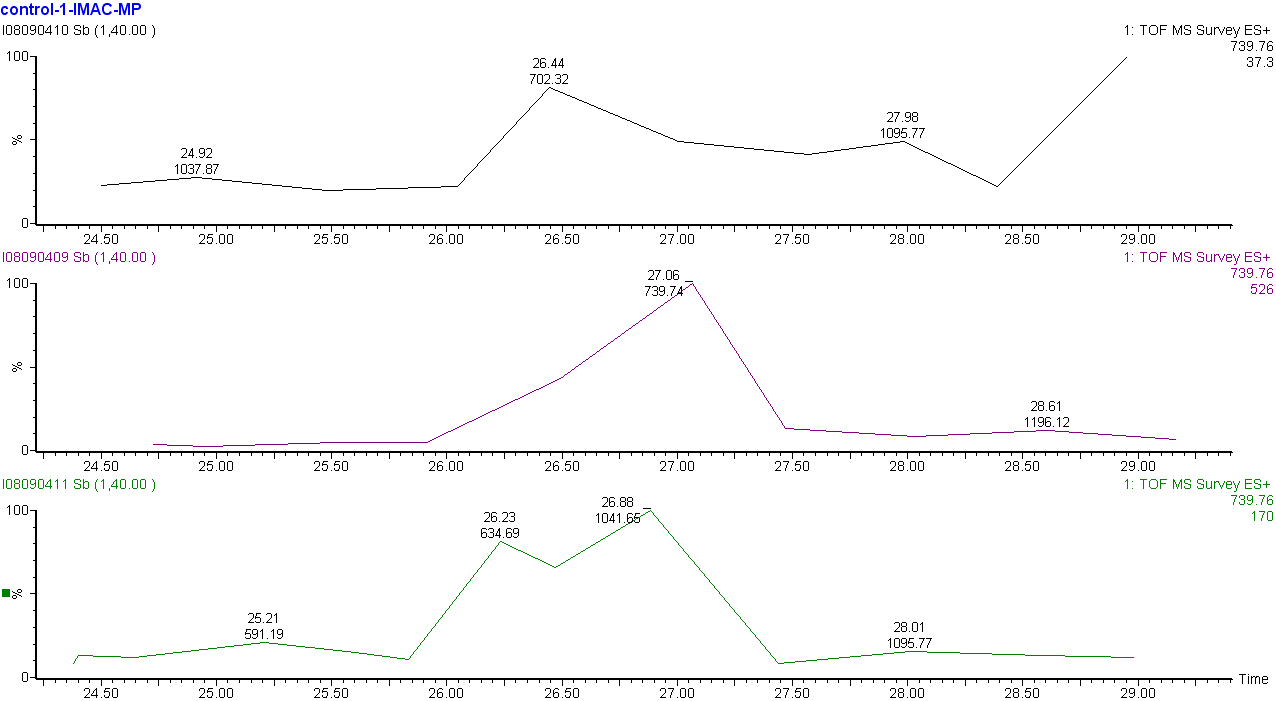


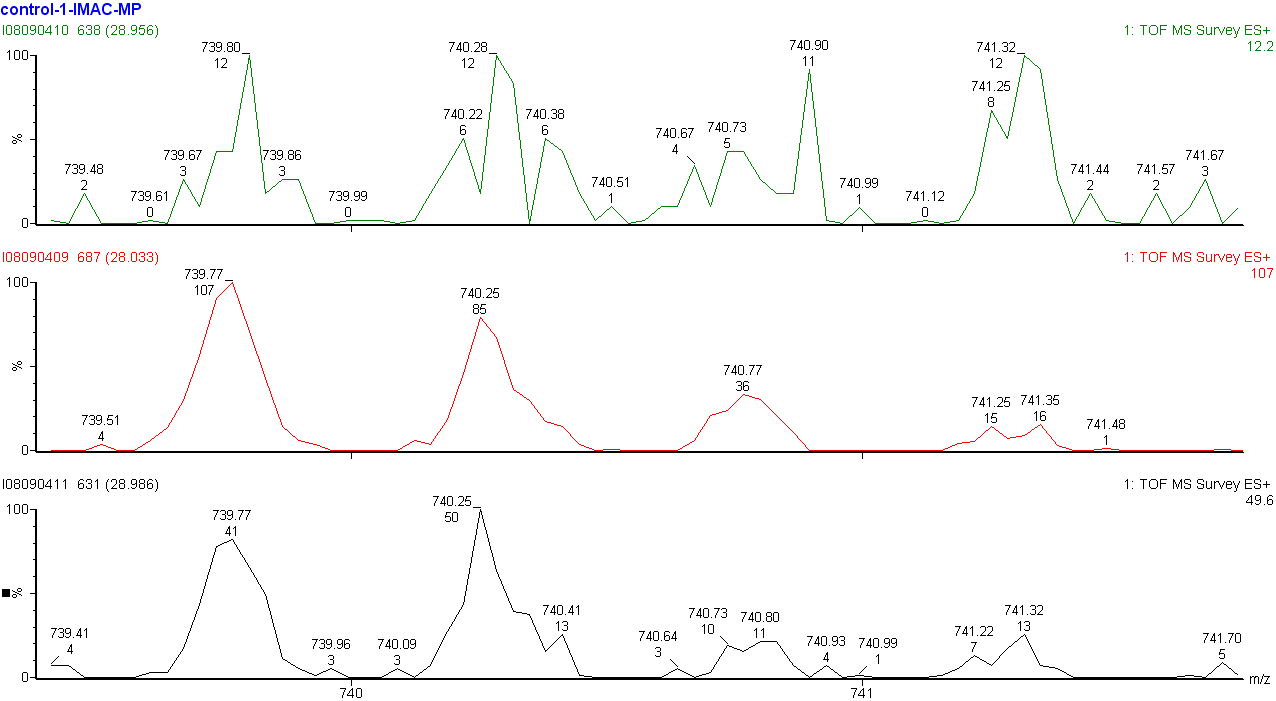


Peak area and MS intensity raw data of Patellin

EILQSEpSFKEEGYLASELQEAEK (1^st^ run –SIC chromatogram and MS spectra)


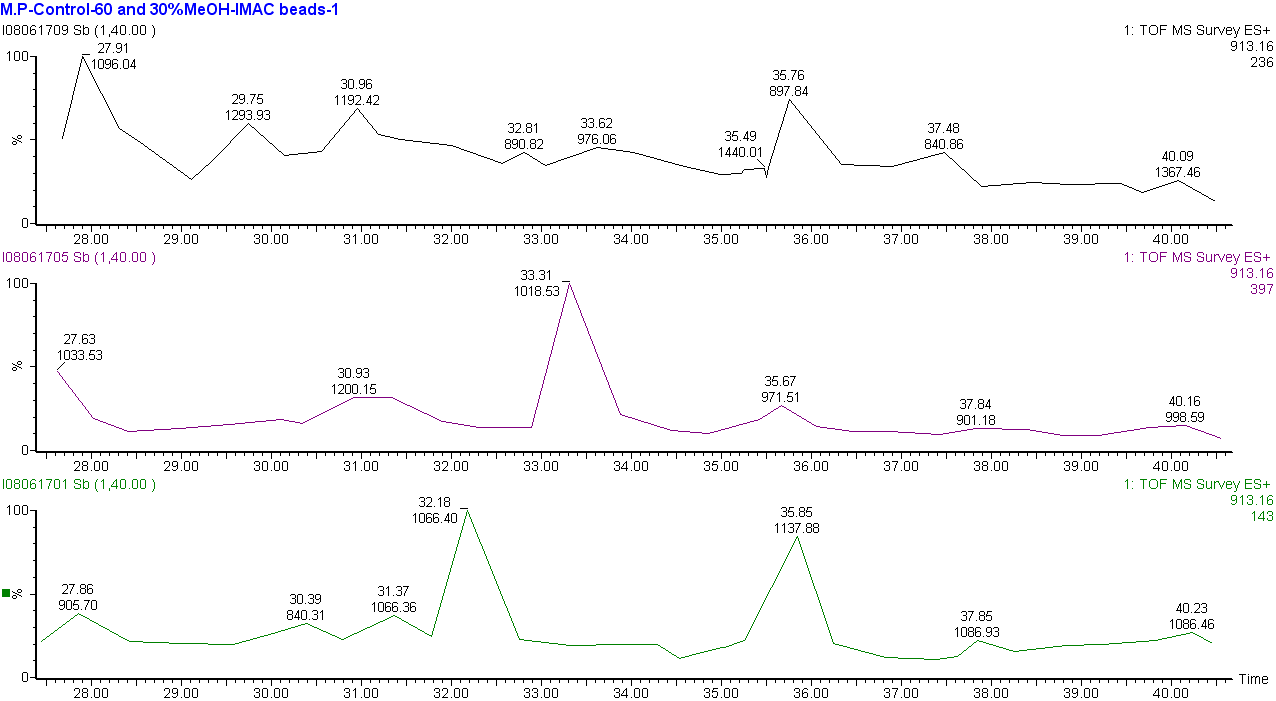


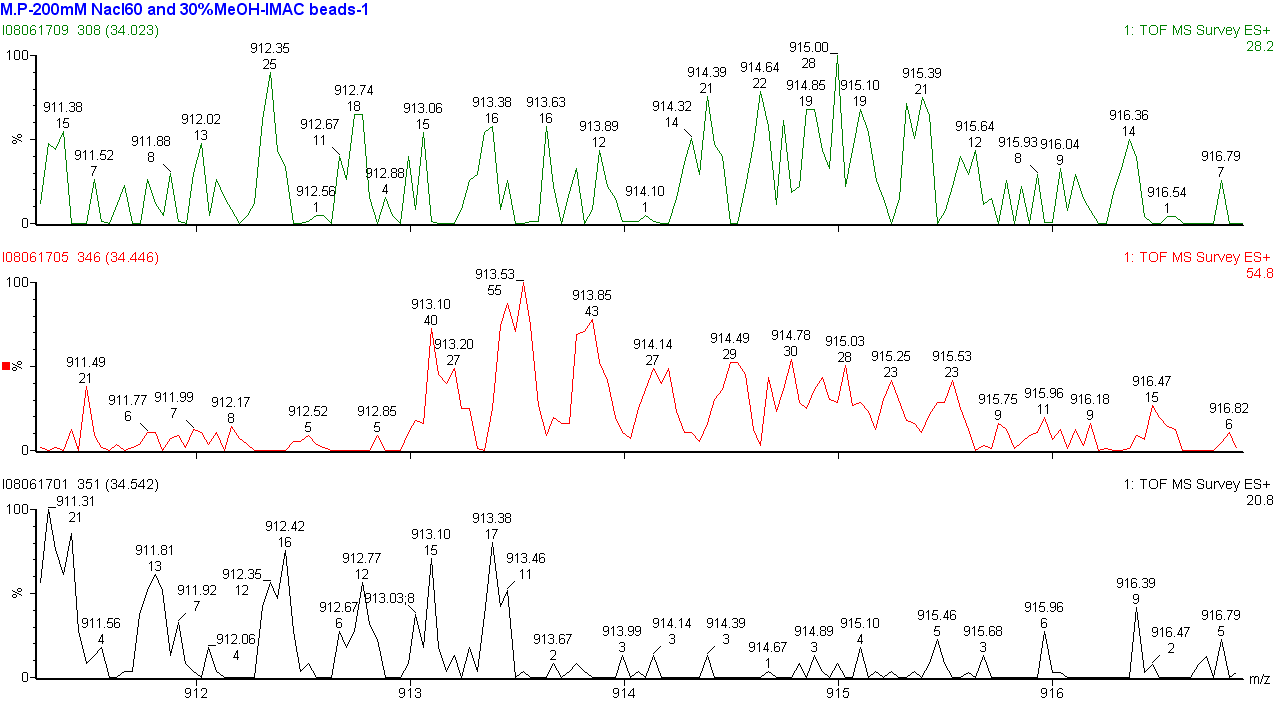


EILQSEpSFKEEGYLASELQEAEK (2^nd^ run –SIC chromatogram and MS spectra)


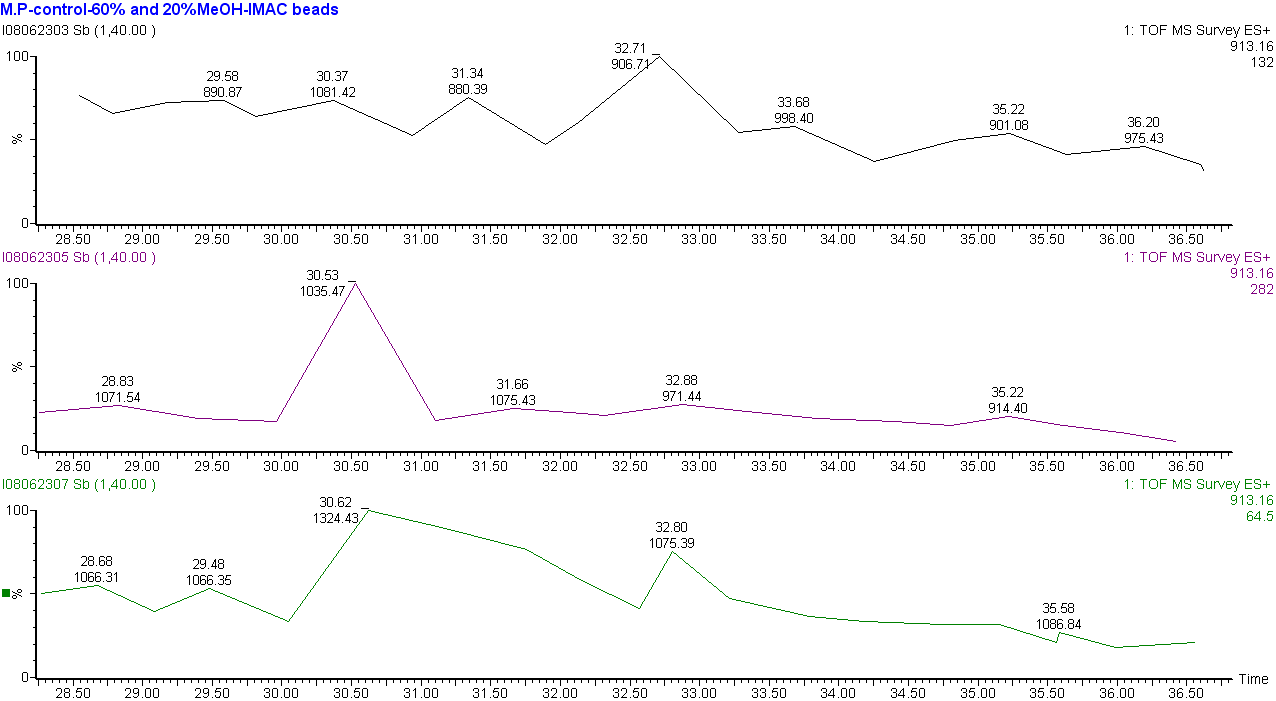


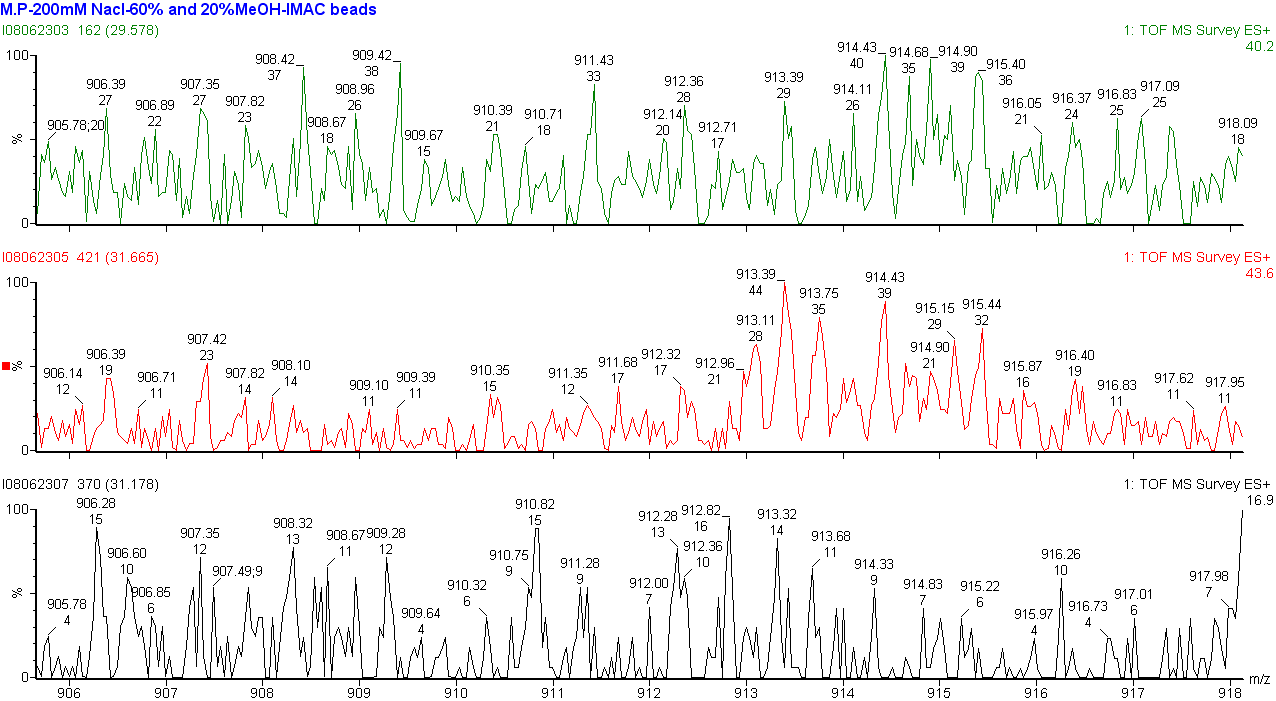


Peak area and MS intensity raw data of **Probable aquaporin PIP2;4**

ALGSFGpSFGSFR (1^st^ run –SIC chromatogram and MS spectra)


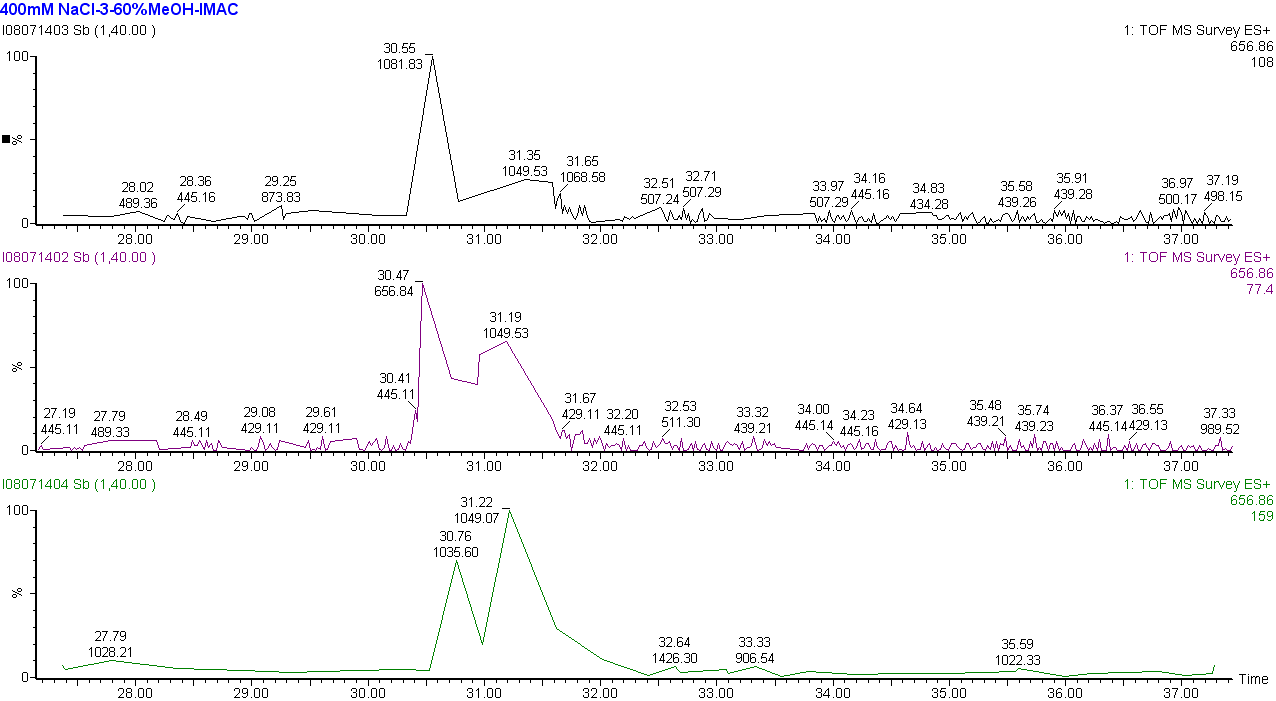


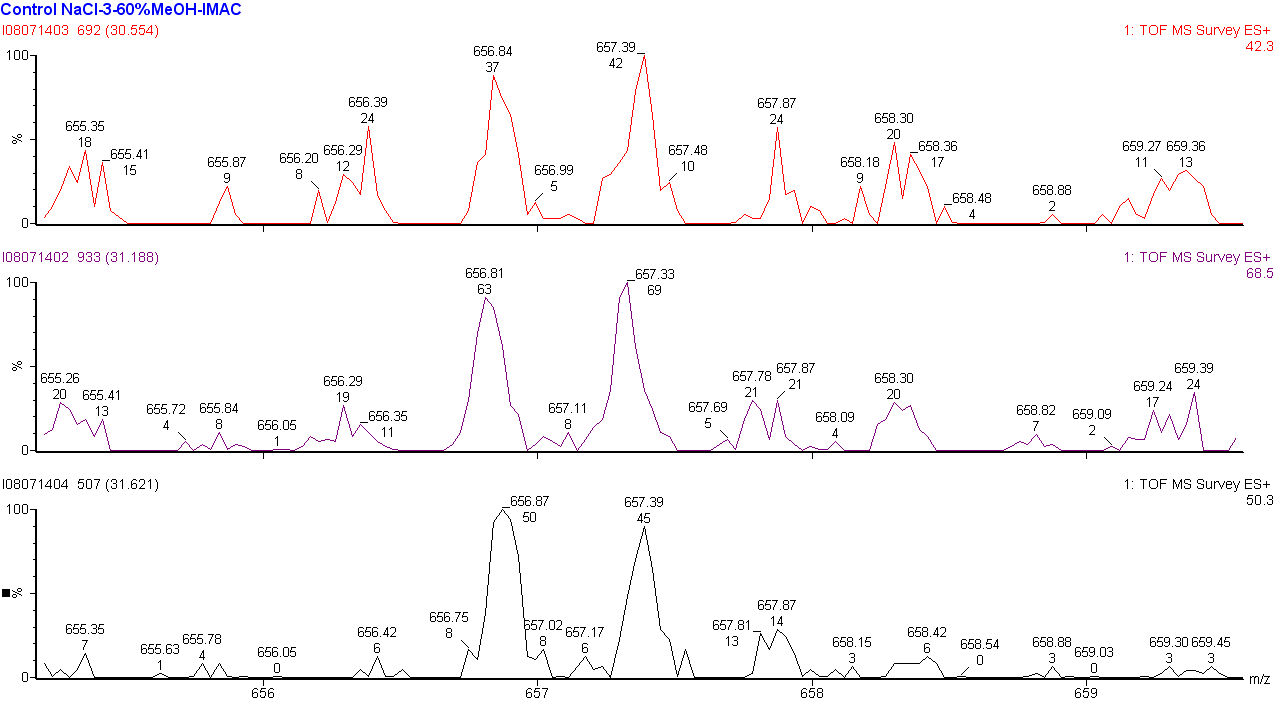


ALGSFGpSFGSFR (2^nd^ run –SIC chromatogram and MS spectra)


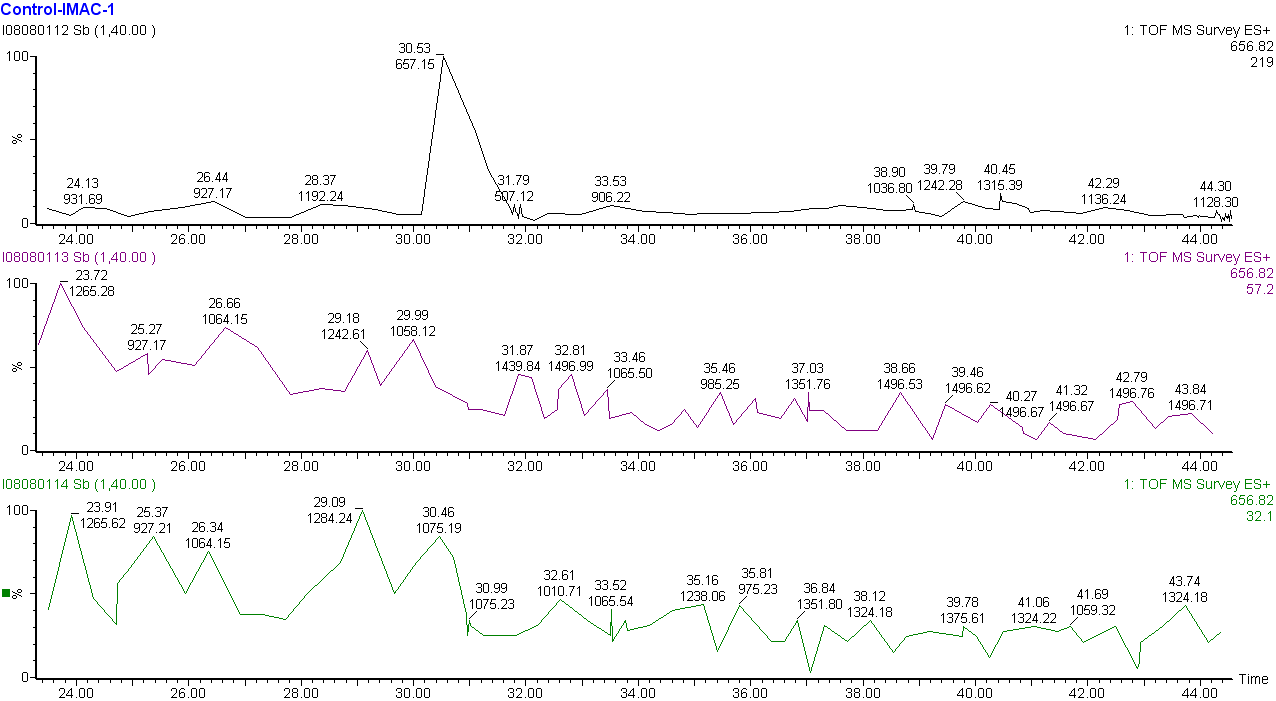


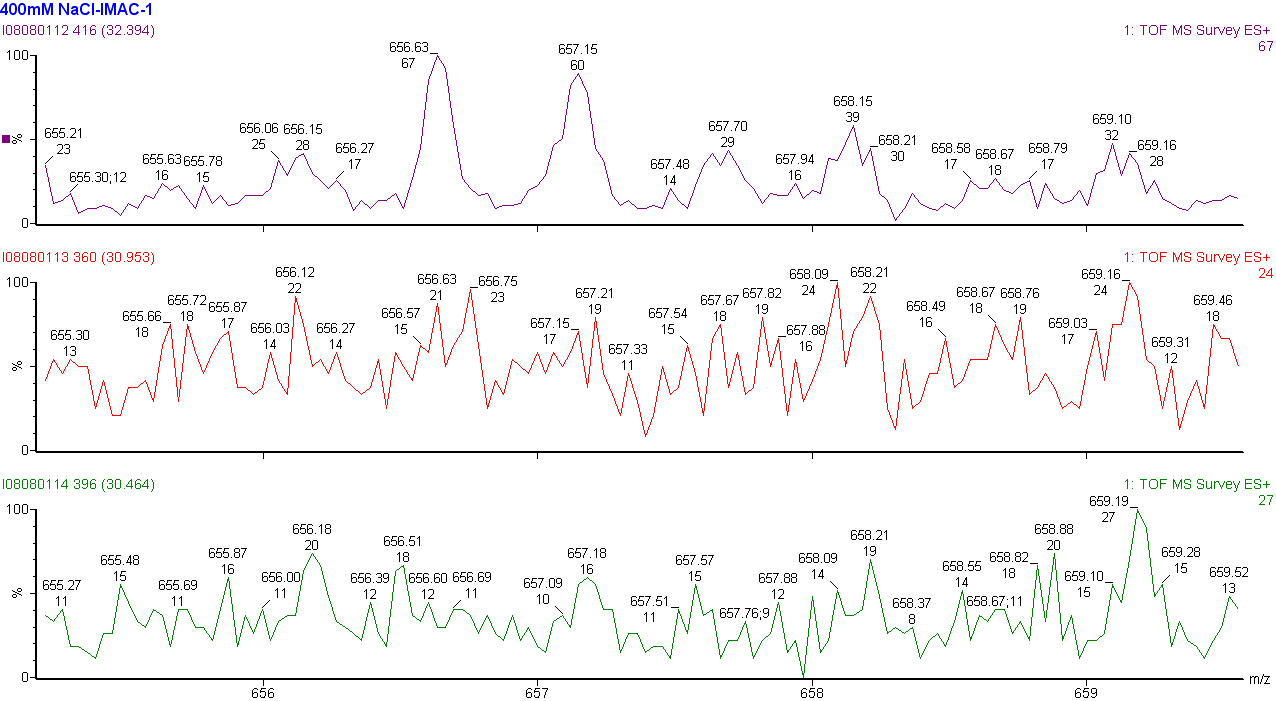


Peak area and MS intensity raw data of **Probable aquaporin PIP2;4**

ALGSFGpSFGpSFR (1^st^ run –SIC chromatogram and MS spectra)


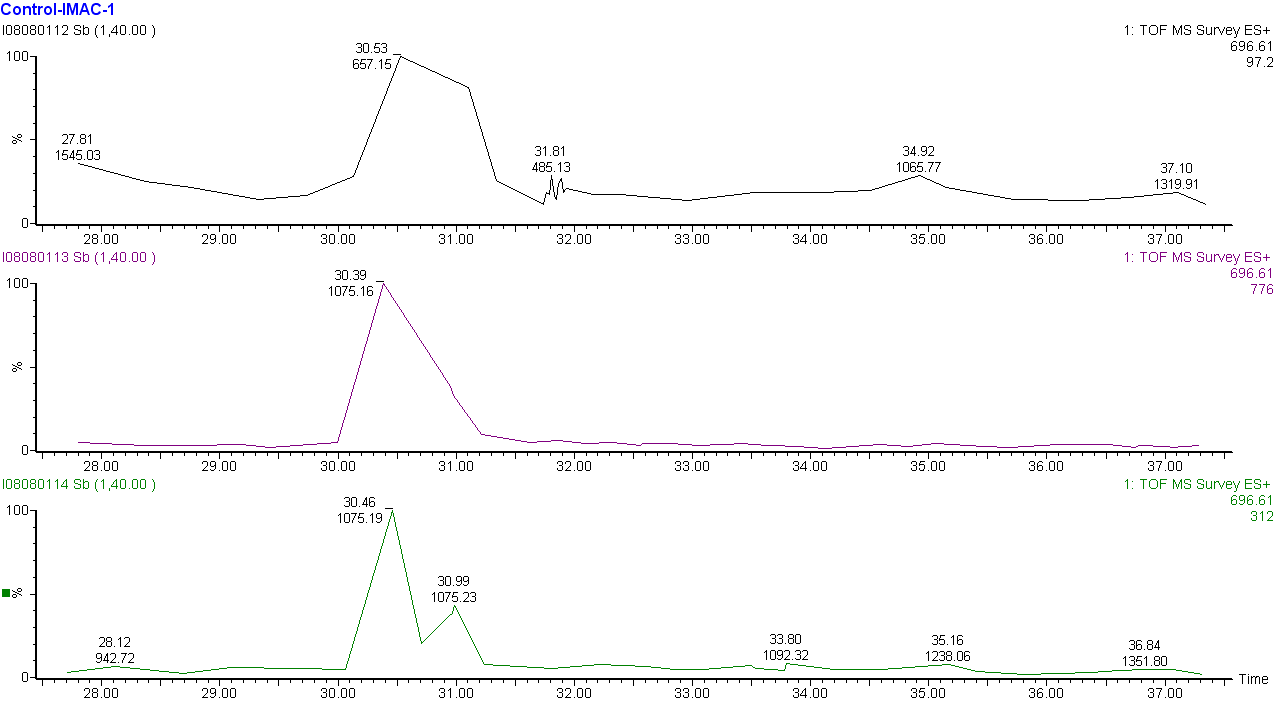


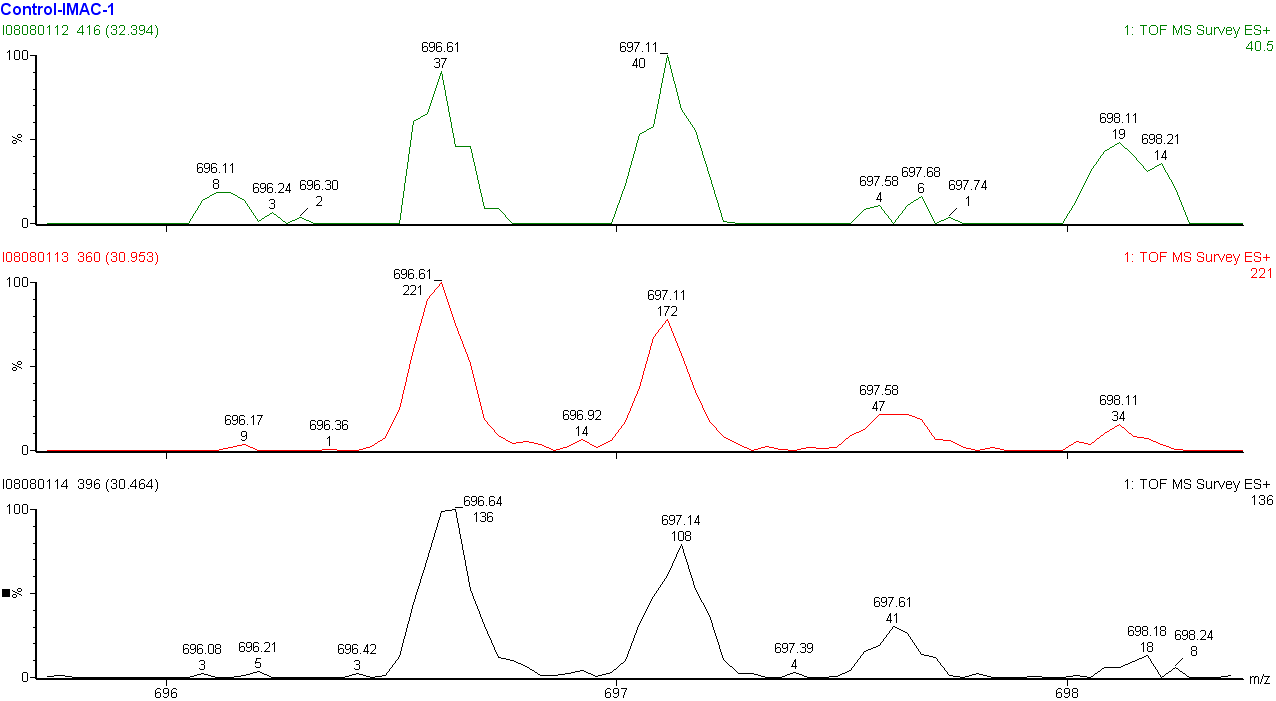


ALGSFGpSFGpSFR (2^nd^ run –SIC chromatogram and MS spectra)


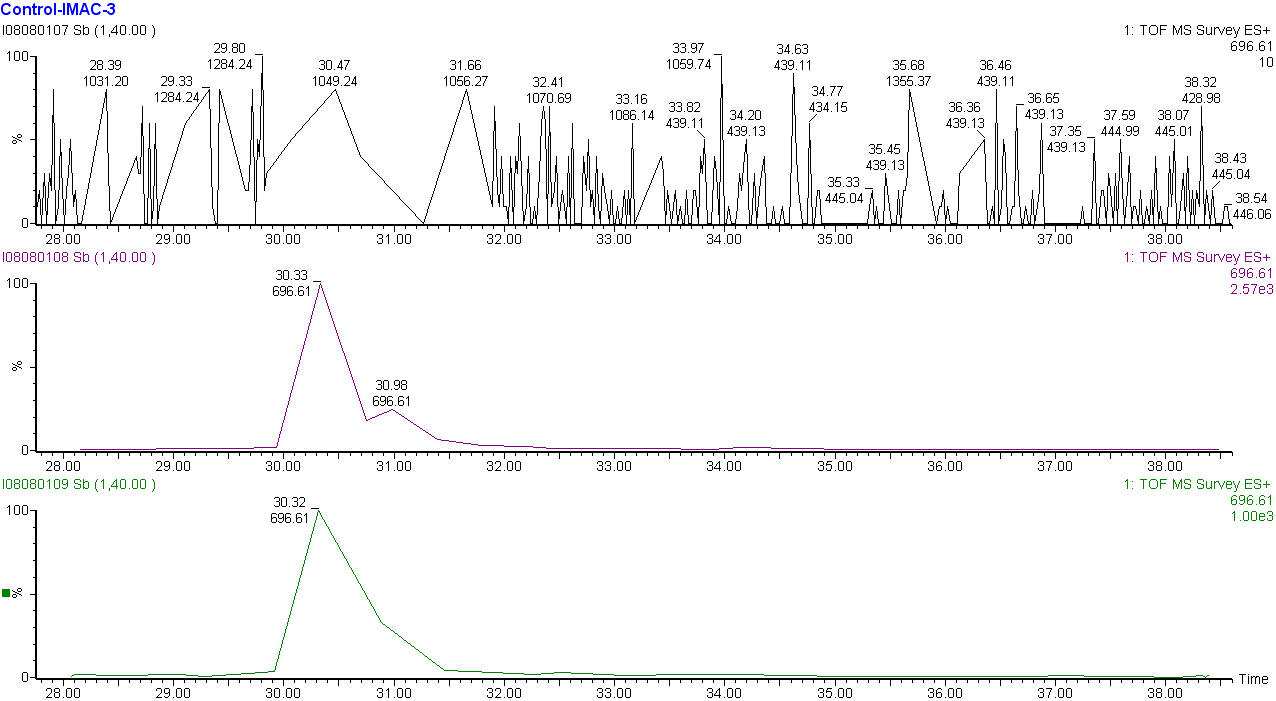


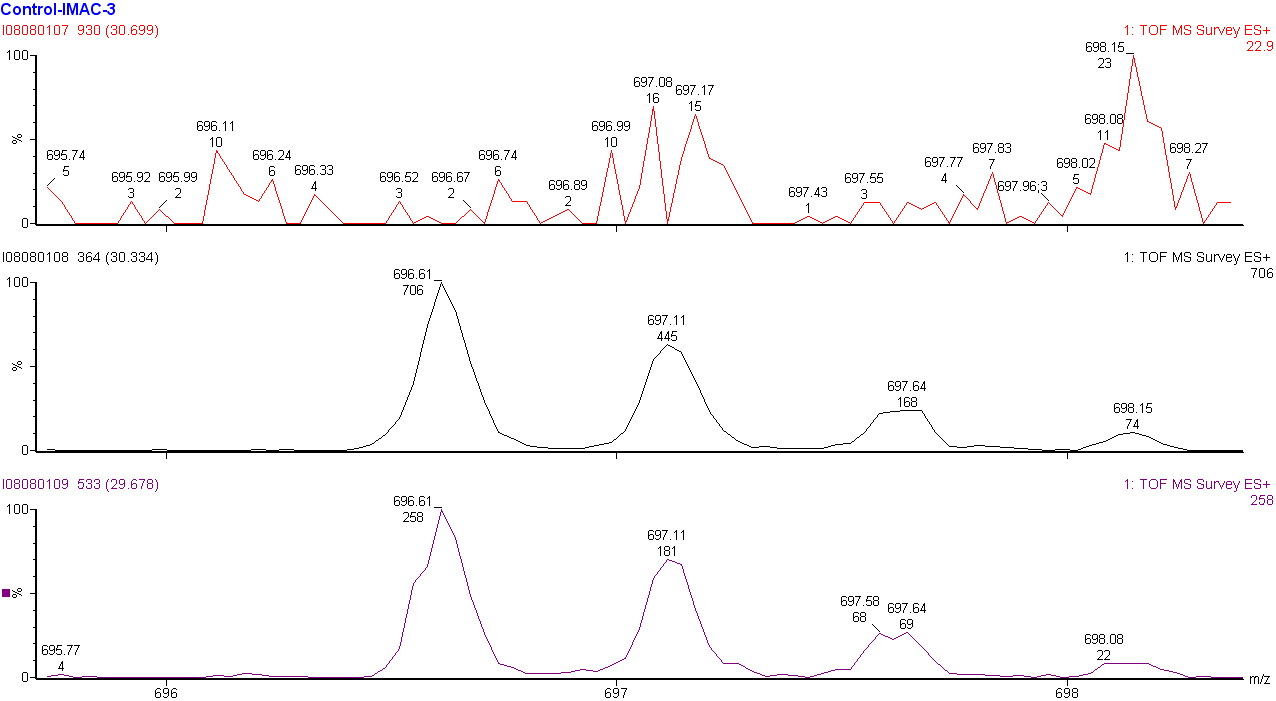


ALGSFGpSFGpSFR (3^rd^ run –SIC chromatogram and MS spectra)


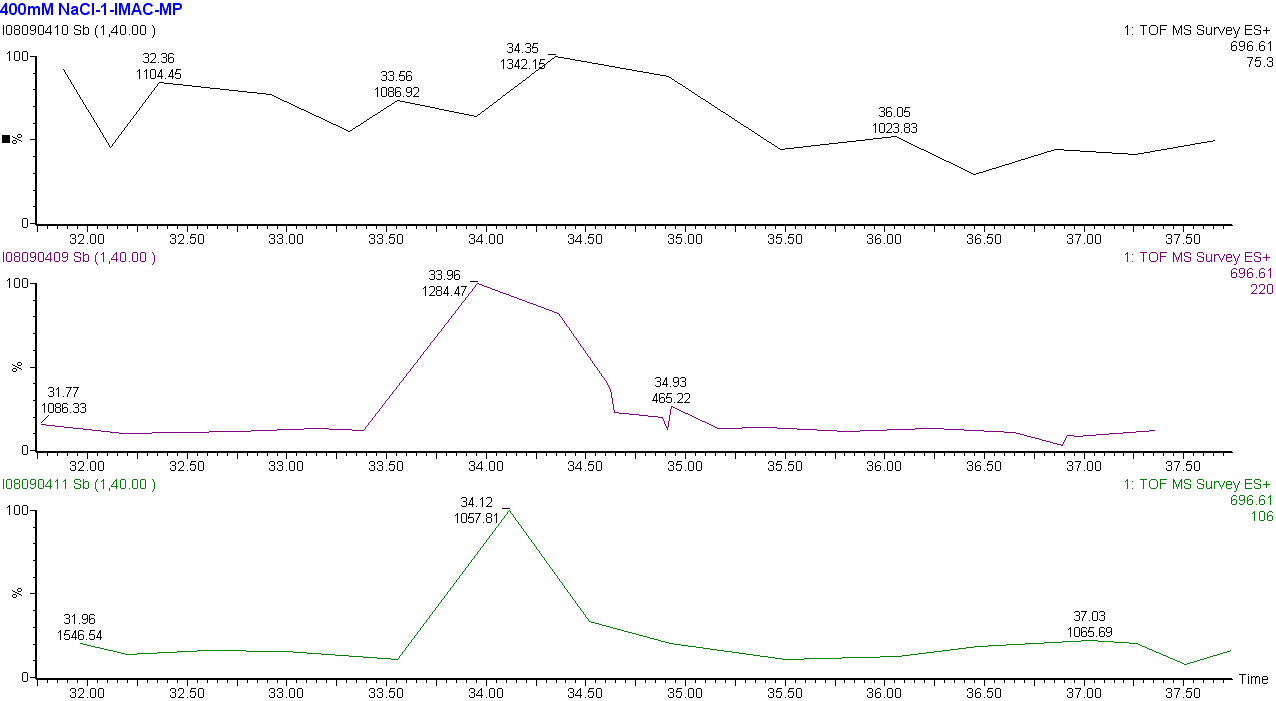


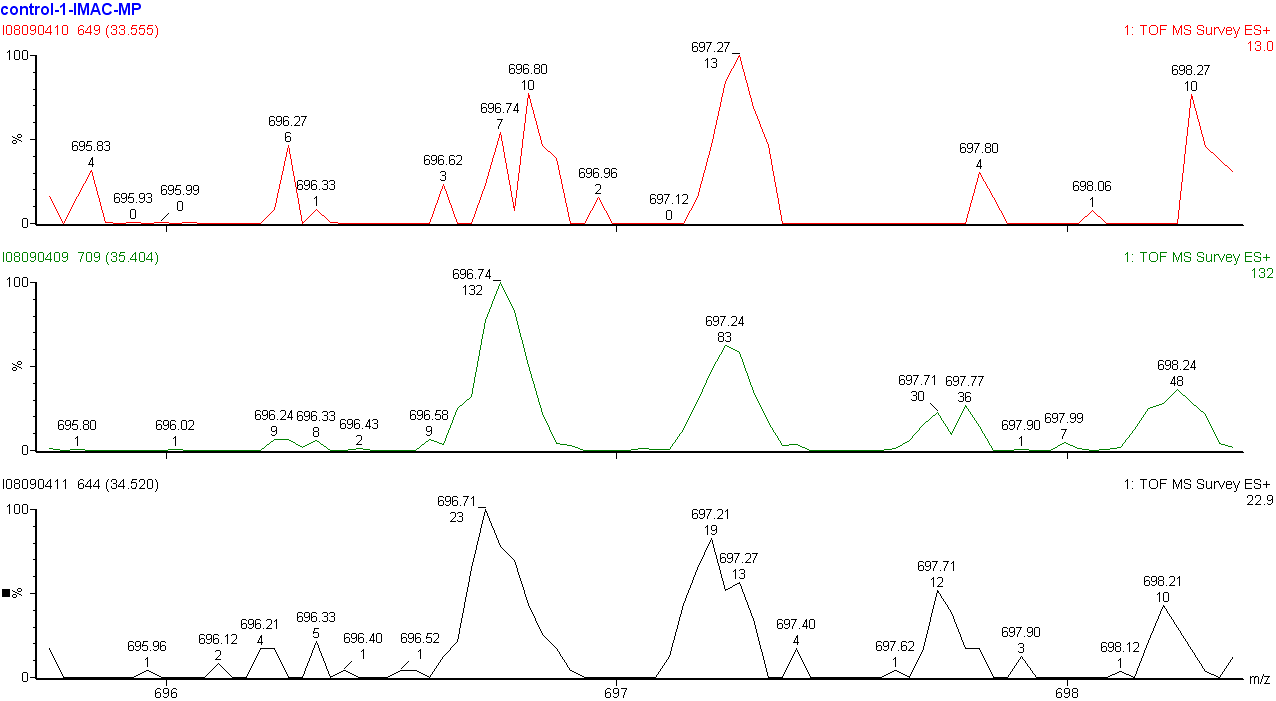


Peak area and MS intensity raw data of **Probable inactive receptor kinase**

LIEEVSHSSGSPNPVpSD (1^st^ run –SIC chromatogram and MS spectra)


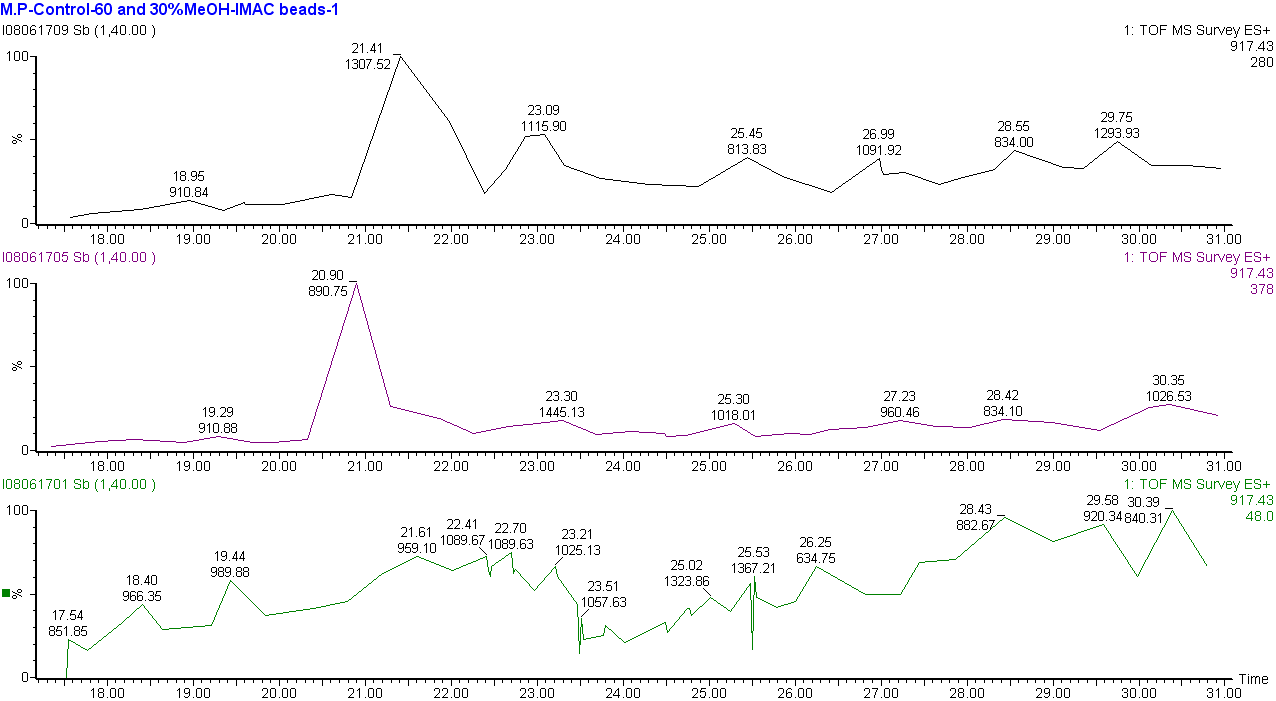


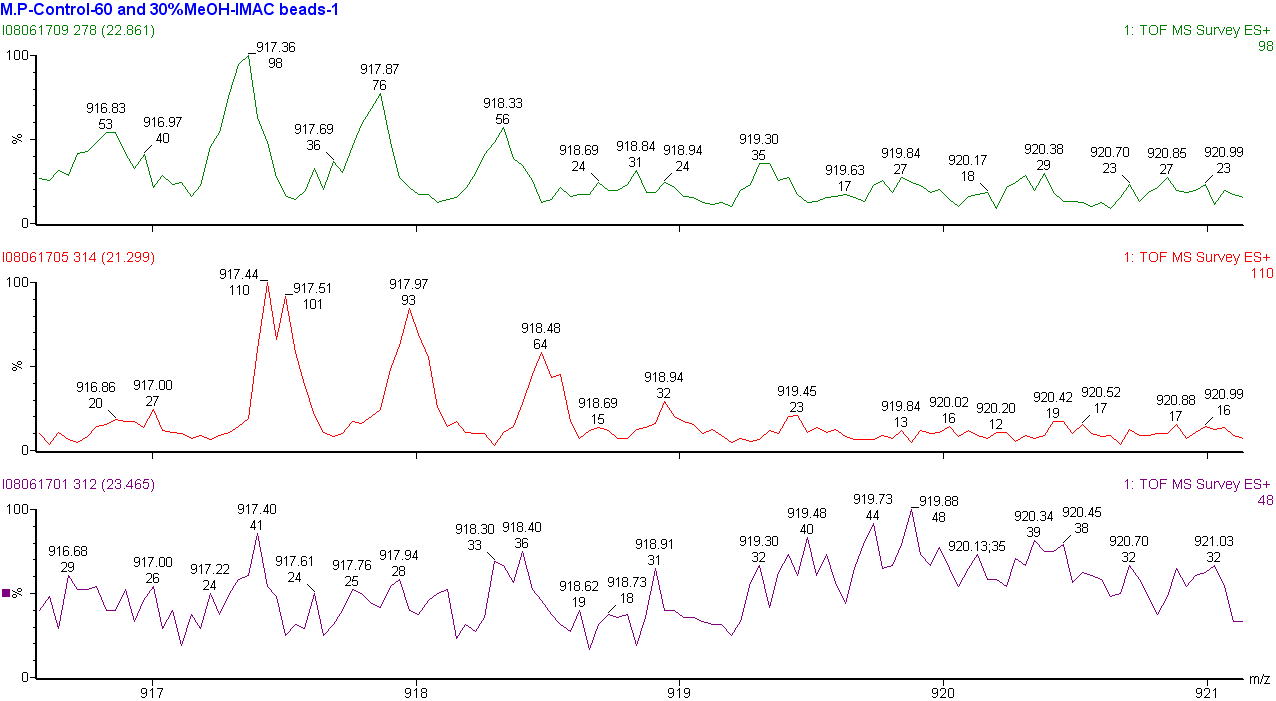


LIEEVSHSSGSPNPVpSD (2^nd^ run –SIC chromatogram and MS spectra)


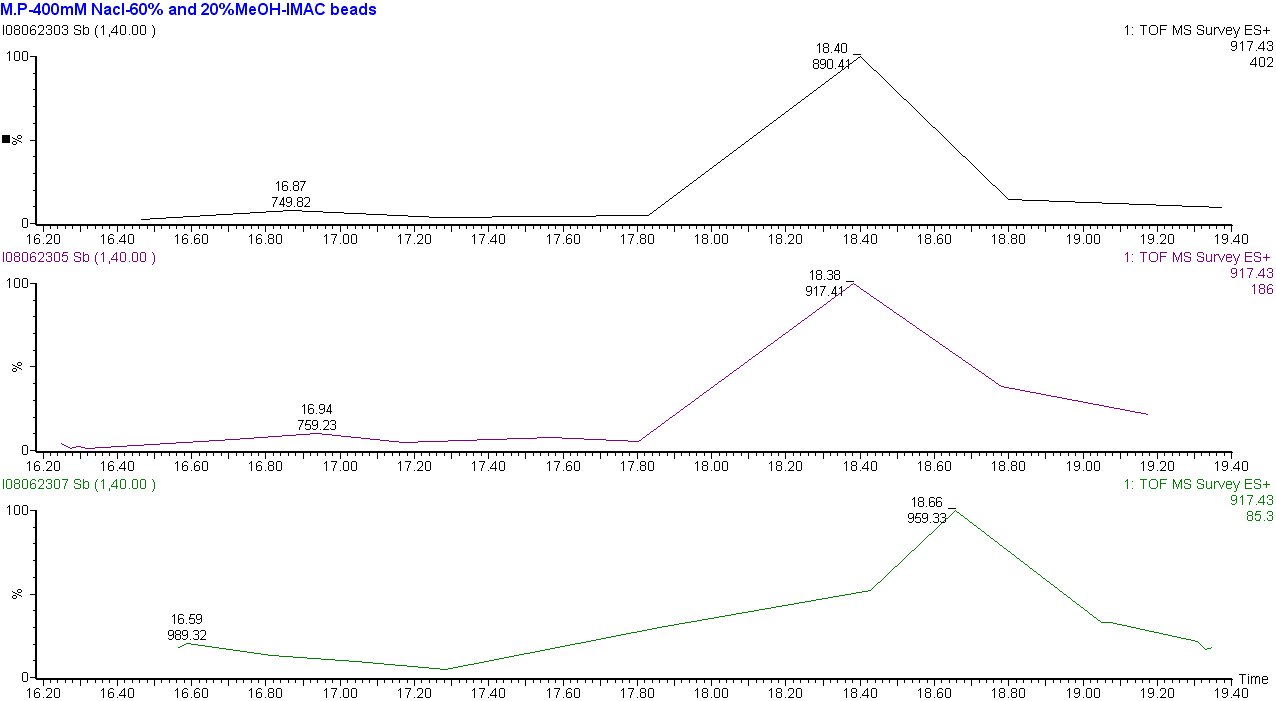


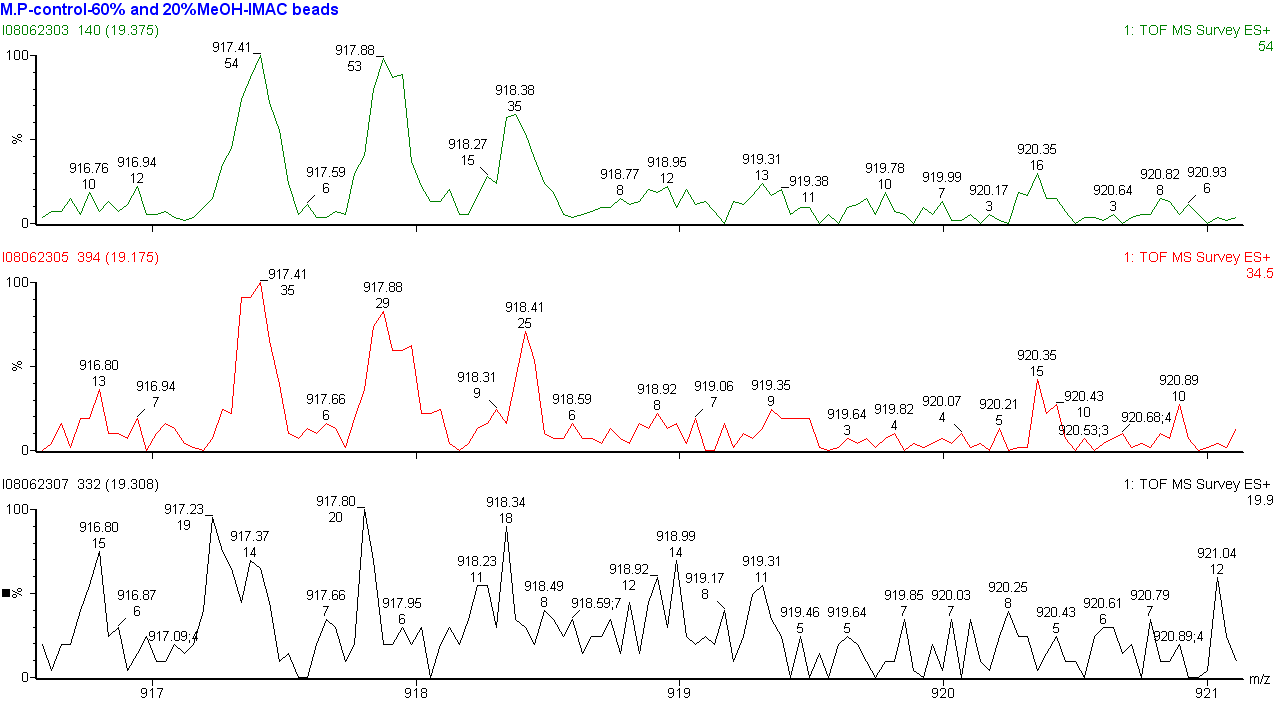


LIEEVSHSSGSPNPVpSD (3^rd^ run –SIC chromatogram and MS spectra)


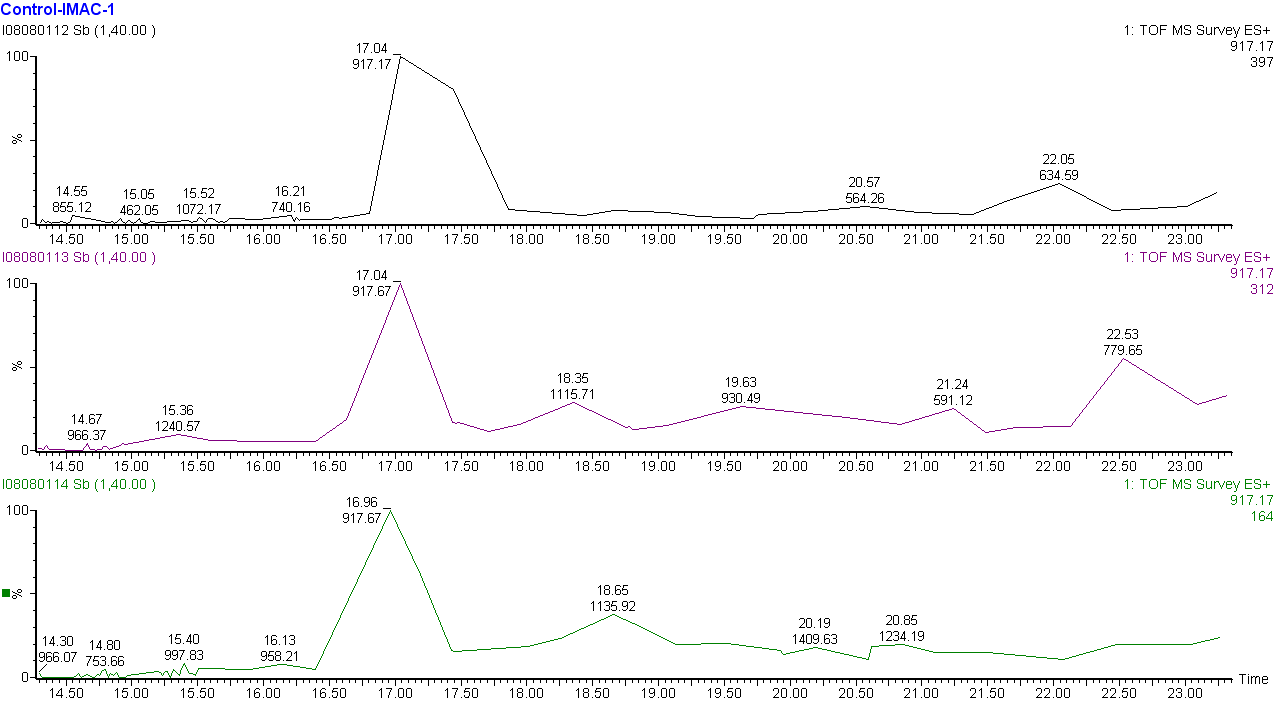


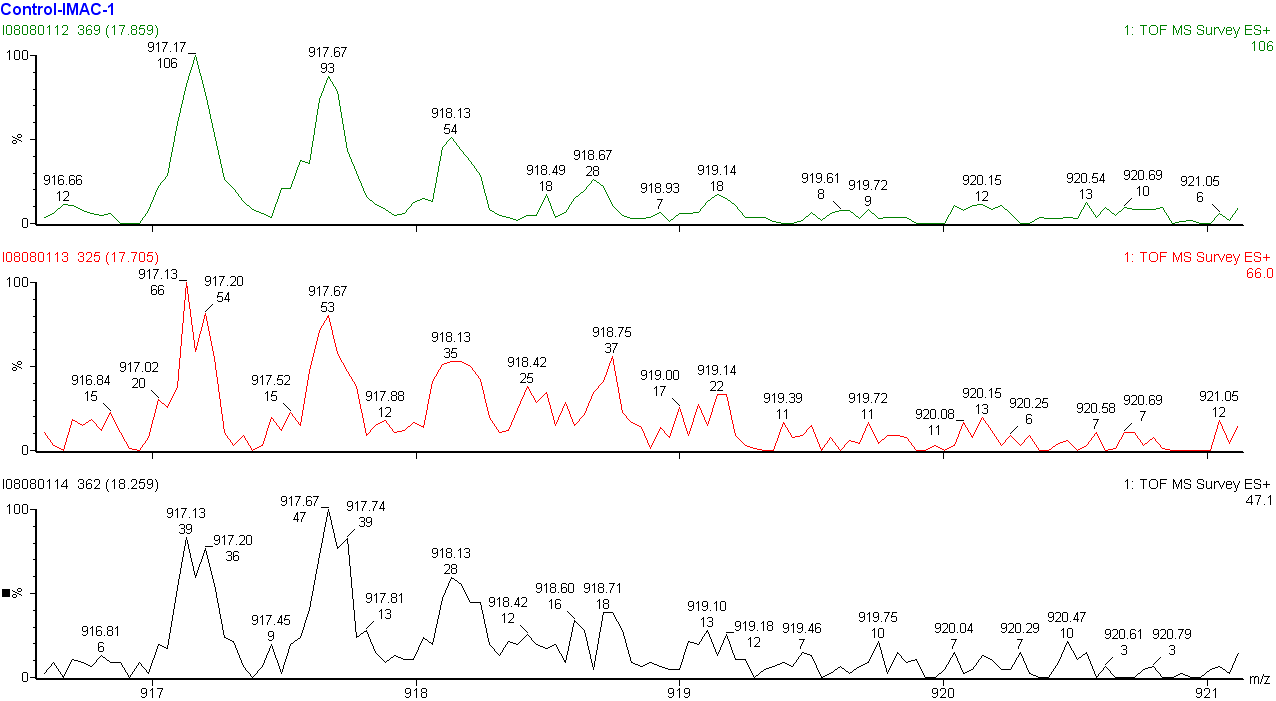


Peak area and MS intensity raw data of **Probable purine permease QTTAEGSANPEPDQILpSPR** (1^st^ run –SIC chromatogram and MS spectra)


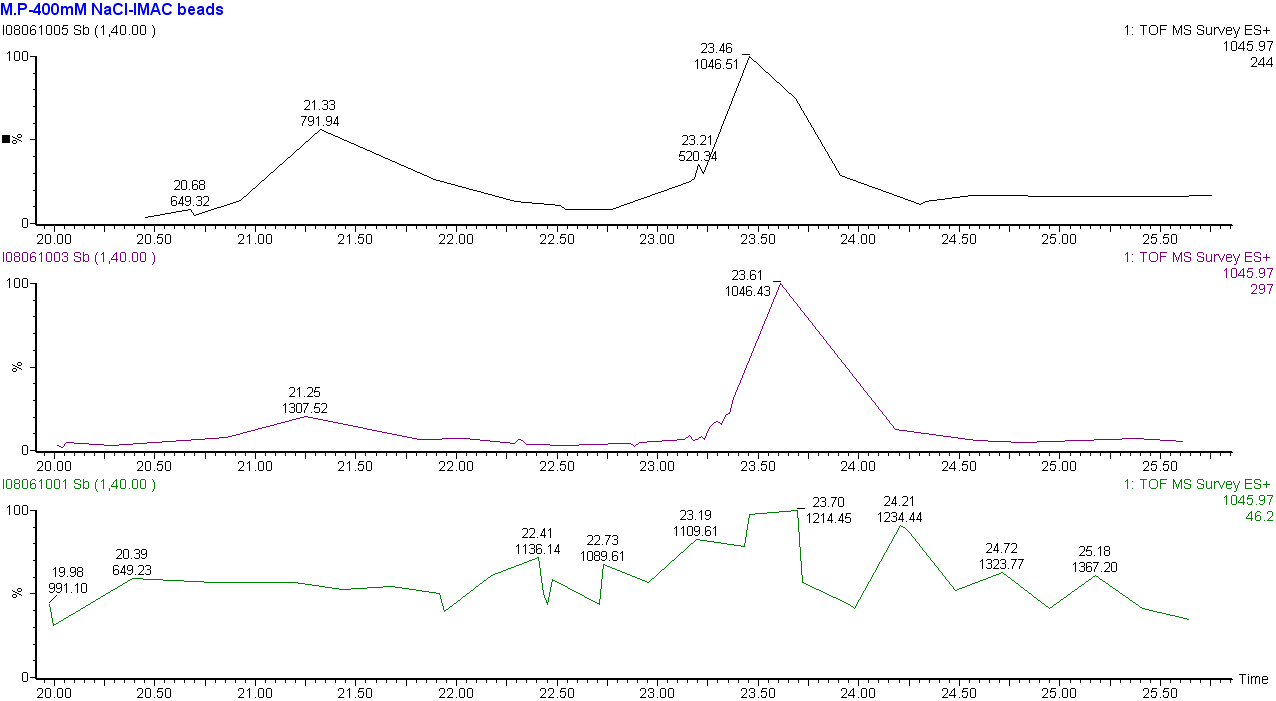


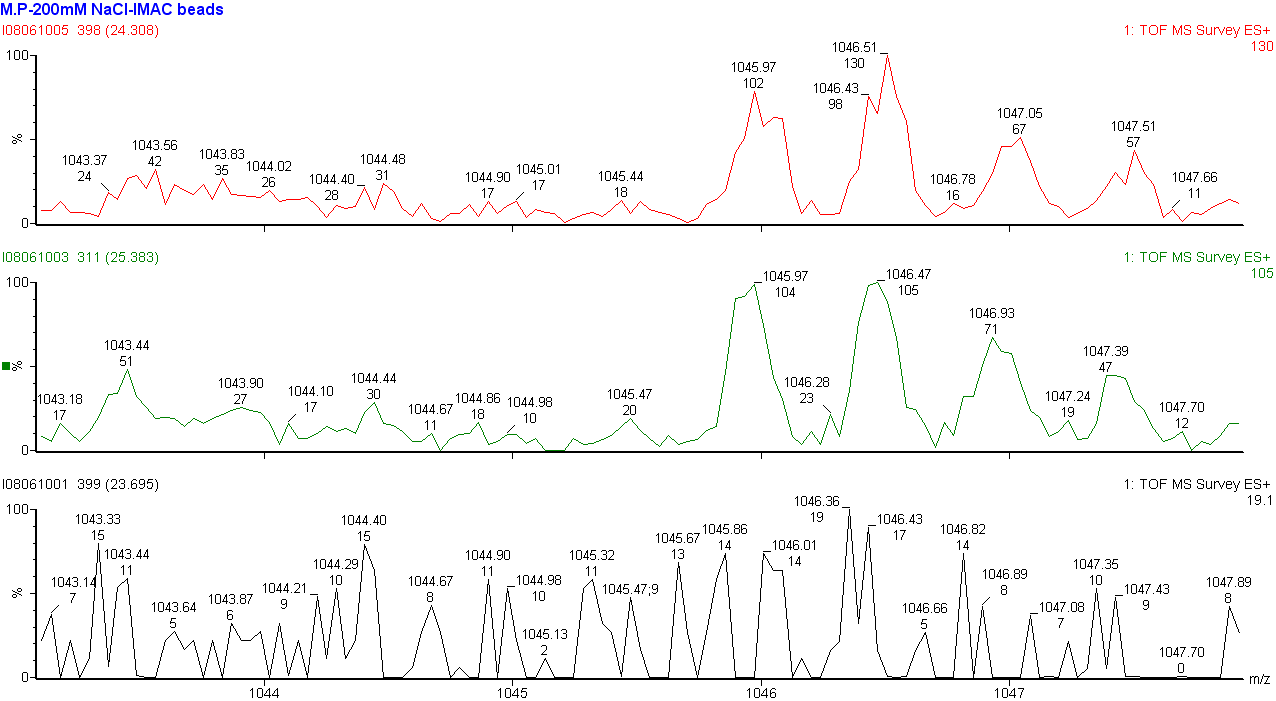


**QTTAEGSANPEPDQILpSPR** (2^nd^ run –SIC chromatogram and MS spectra)


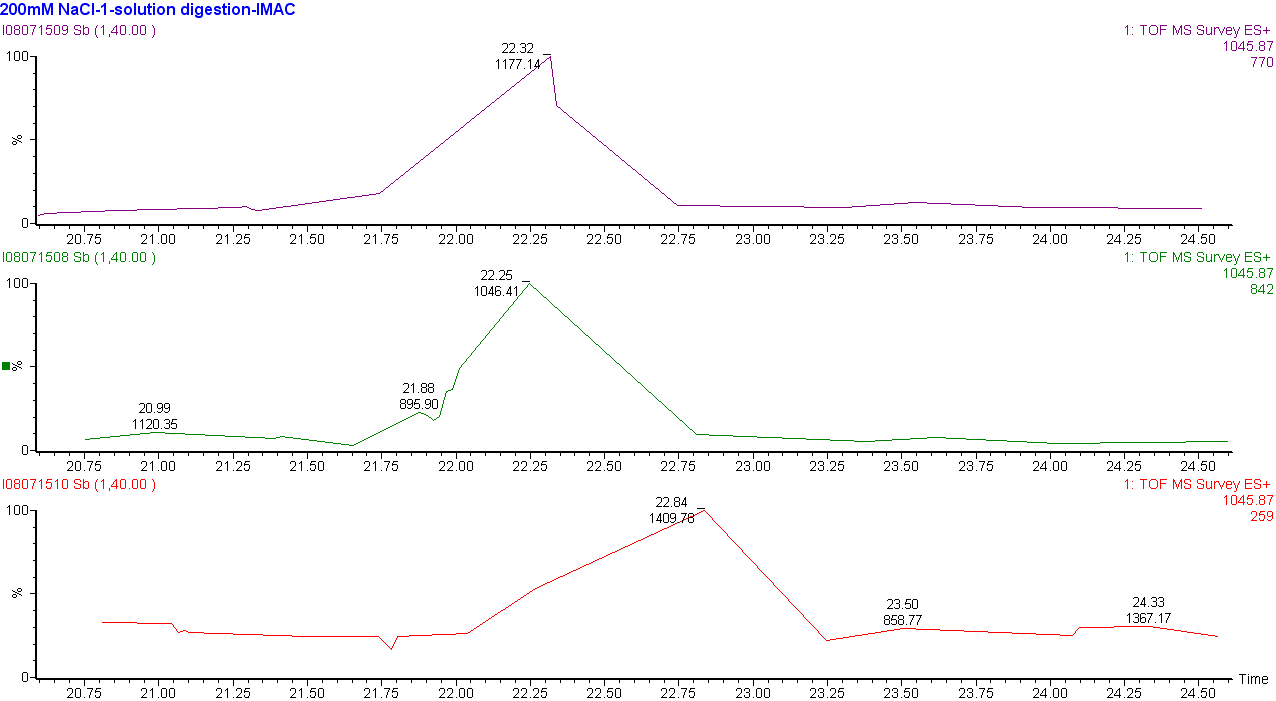


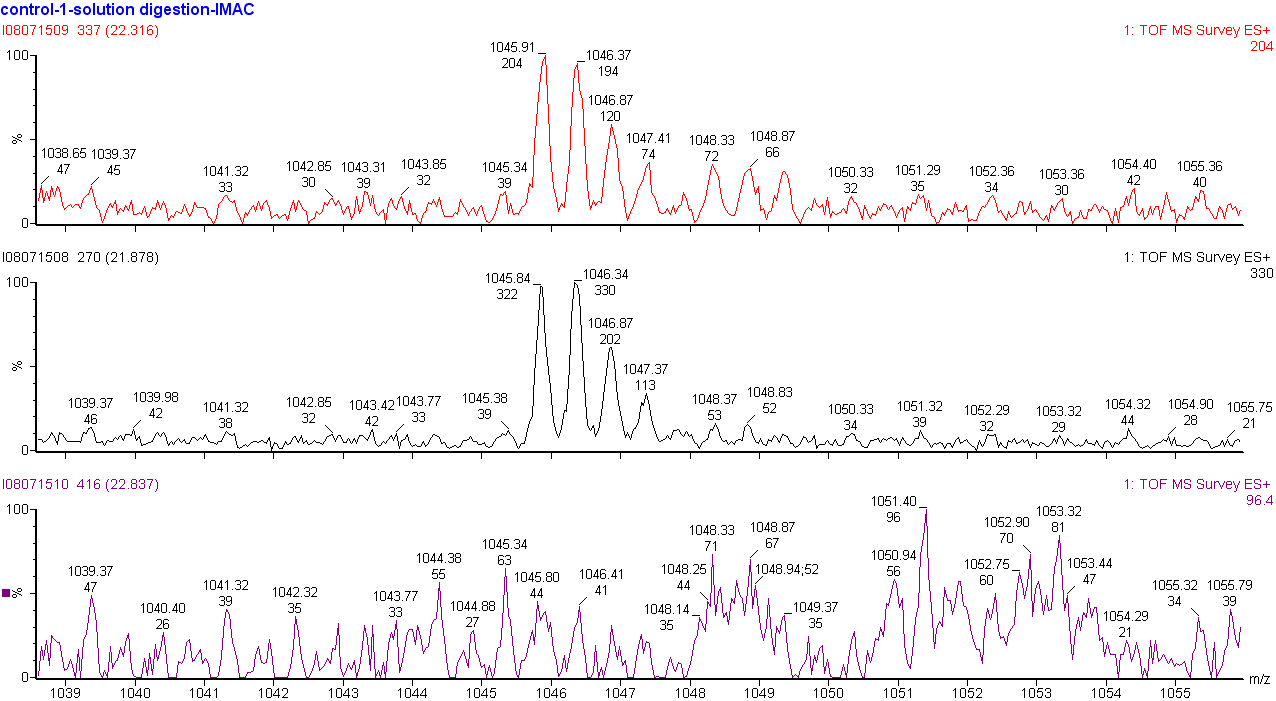


**QTTAEGSANPEPDQILpSPR** (3^rd^ run –SIC chromatogram and MS spectra)


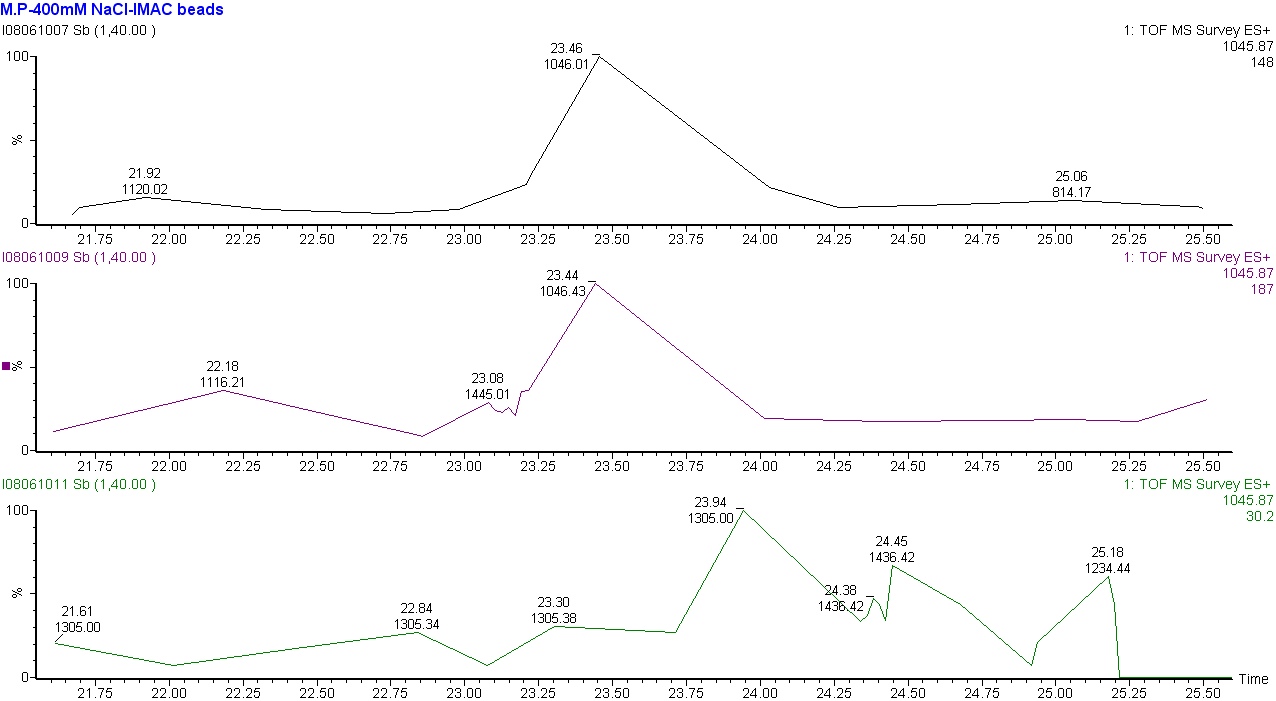


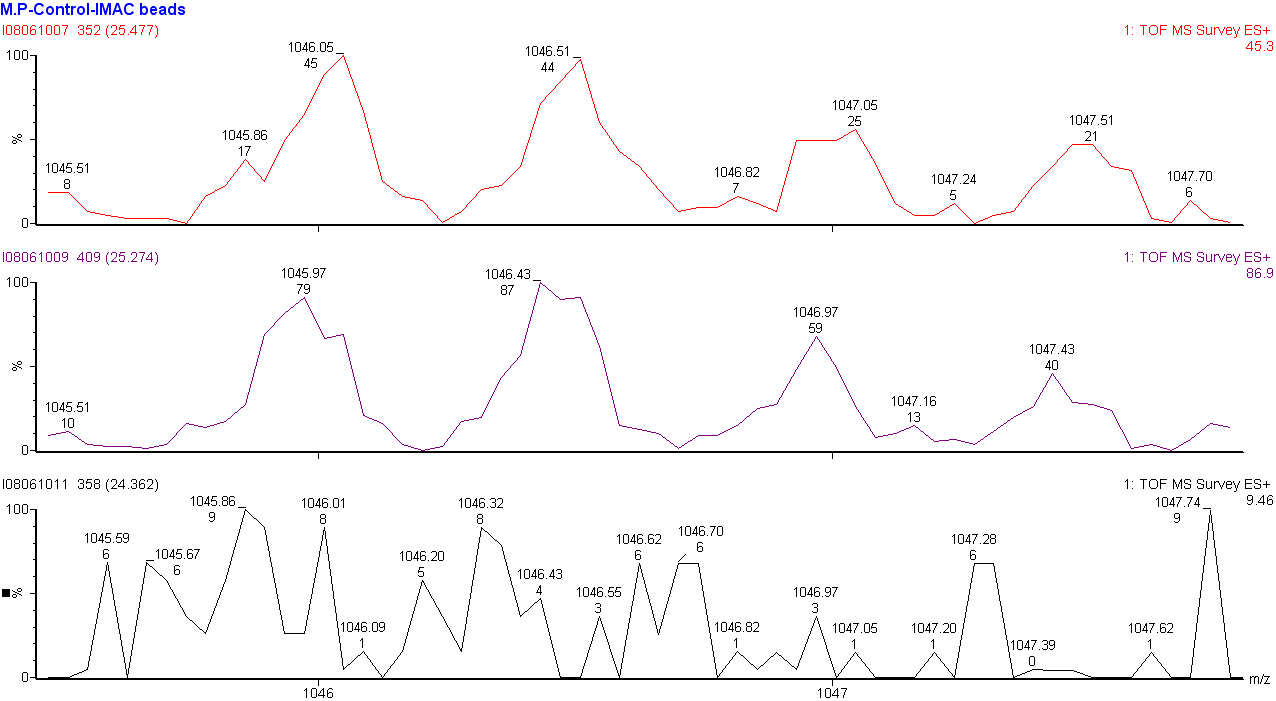


Peak area and MS intensity raw data of **Sugar transport protein 1 GVDDVpSQEFDDLVAASK** (1^st^ run –SIC chromatogram and MS spectra)


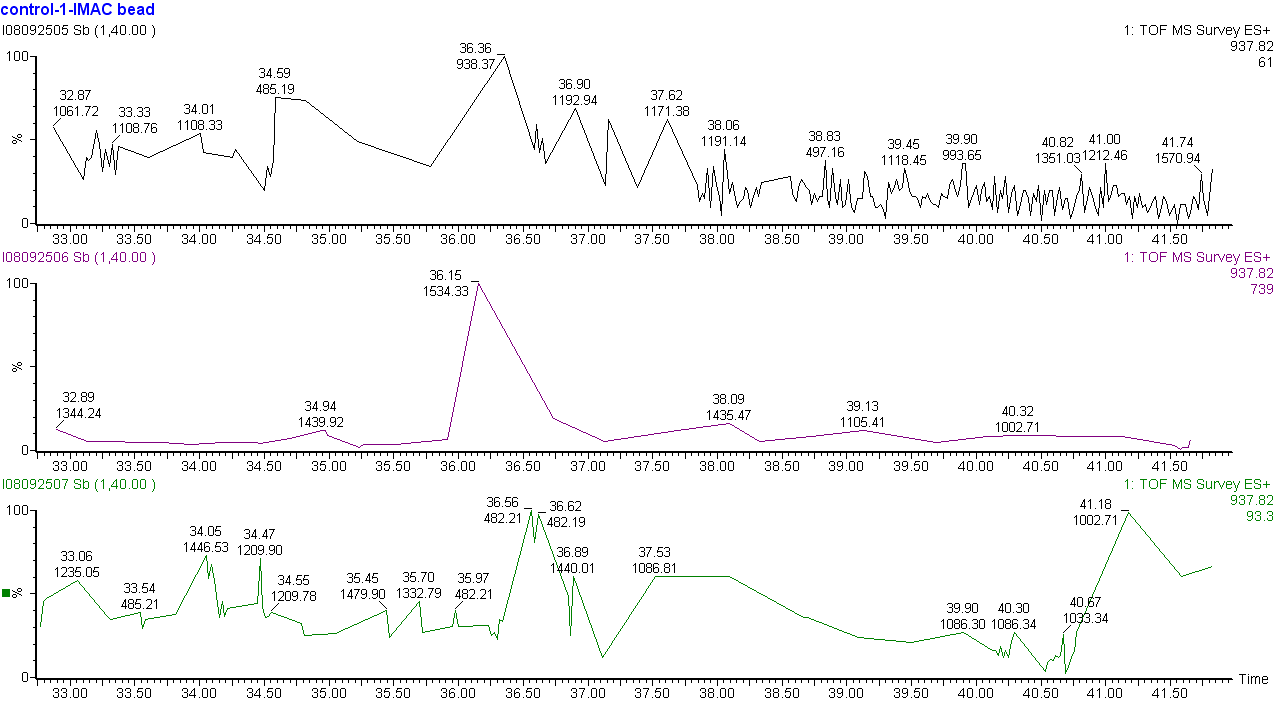


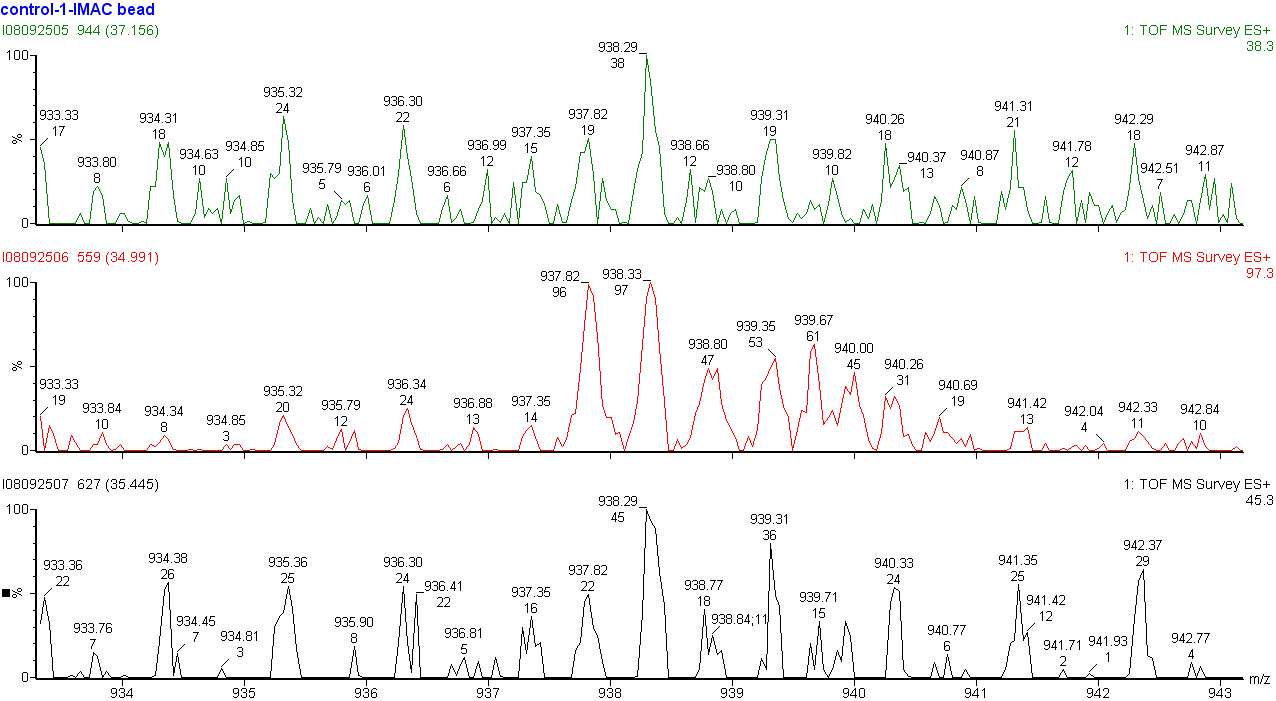


D/H ratio raw data of **Aquaporin PIP2**

***SLGpSFRSAANV**


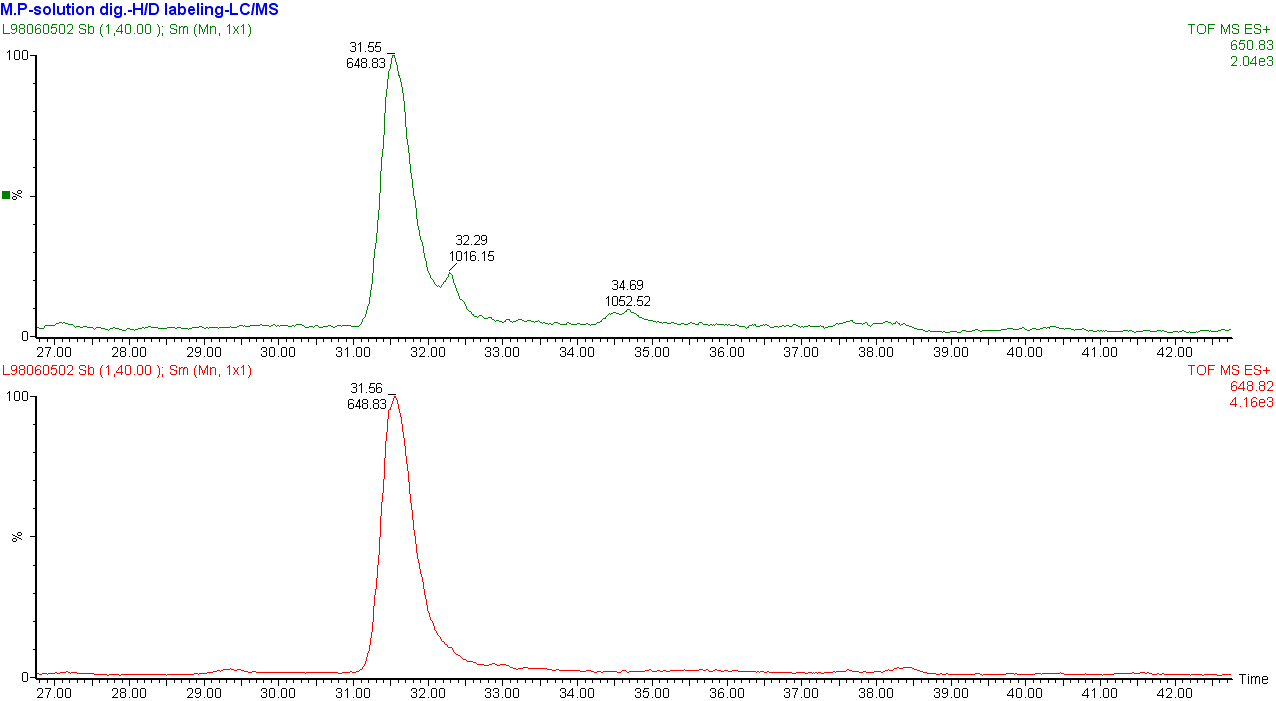


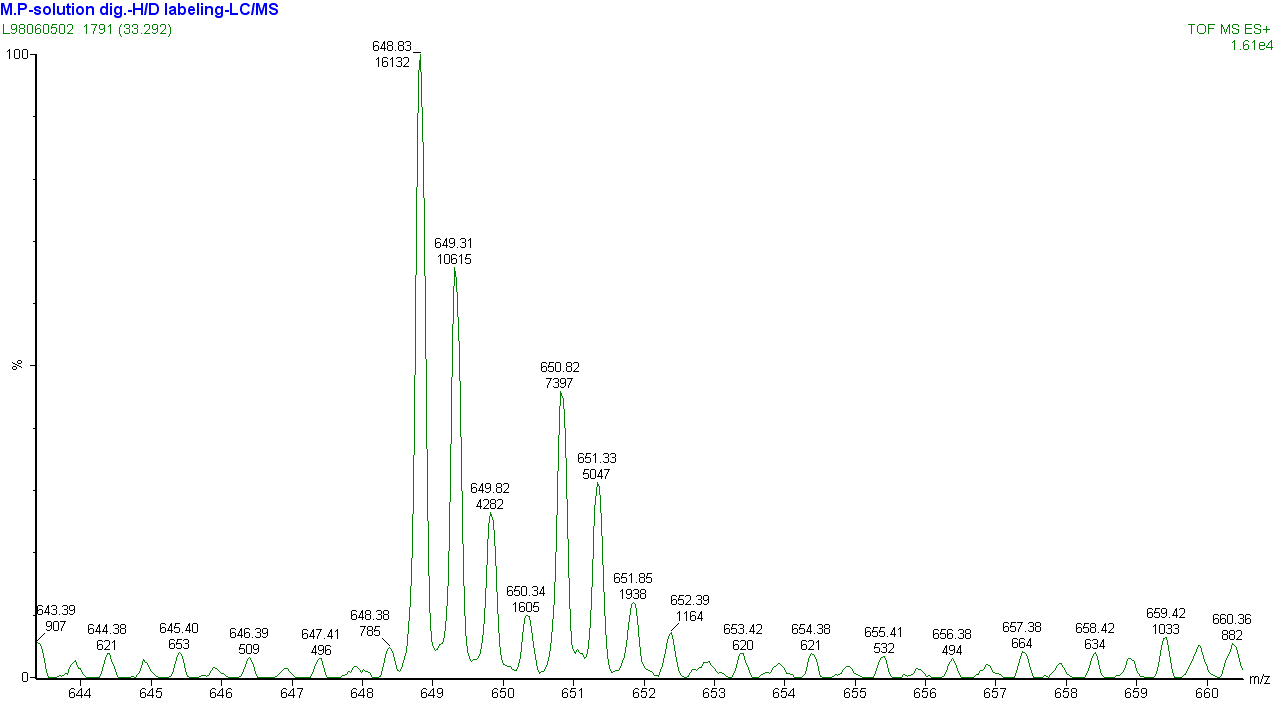


D/H ratio raw data of **FAM10 family protein**

***VEEEEEEDEIVEpSDVELEGDTVEPDNDPPQ*K**


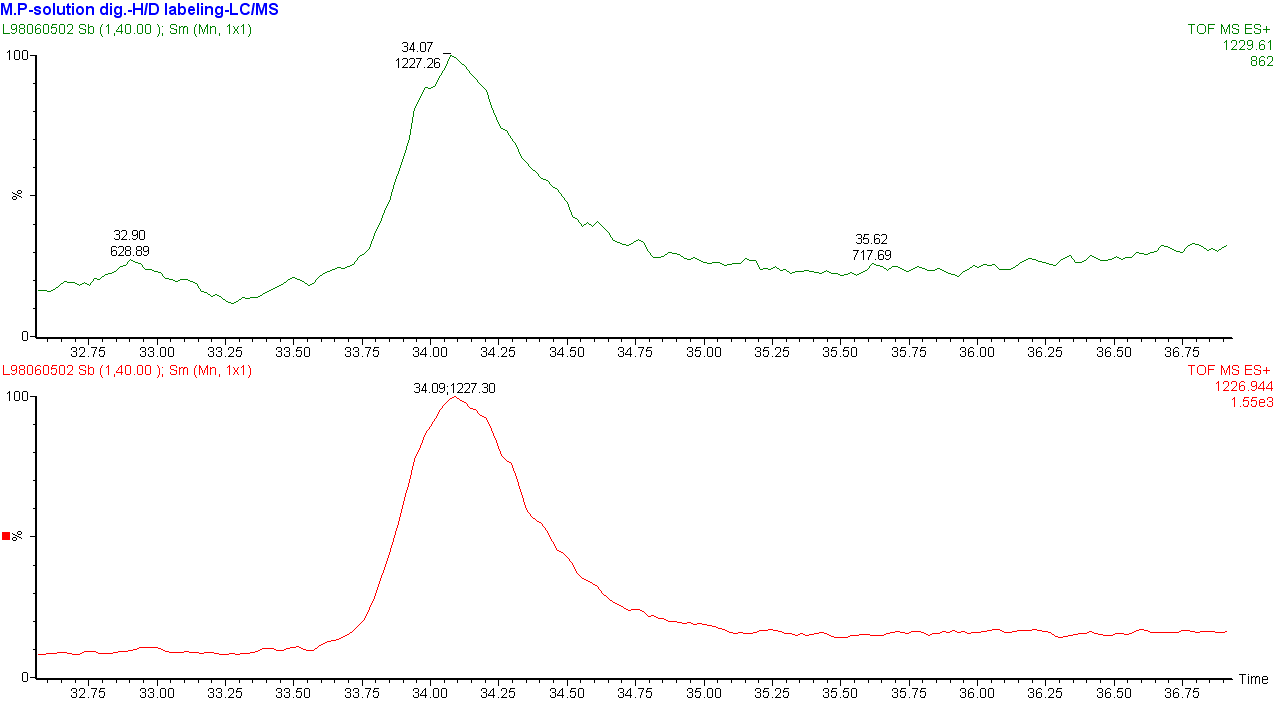


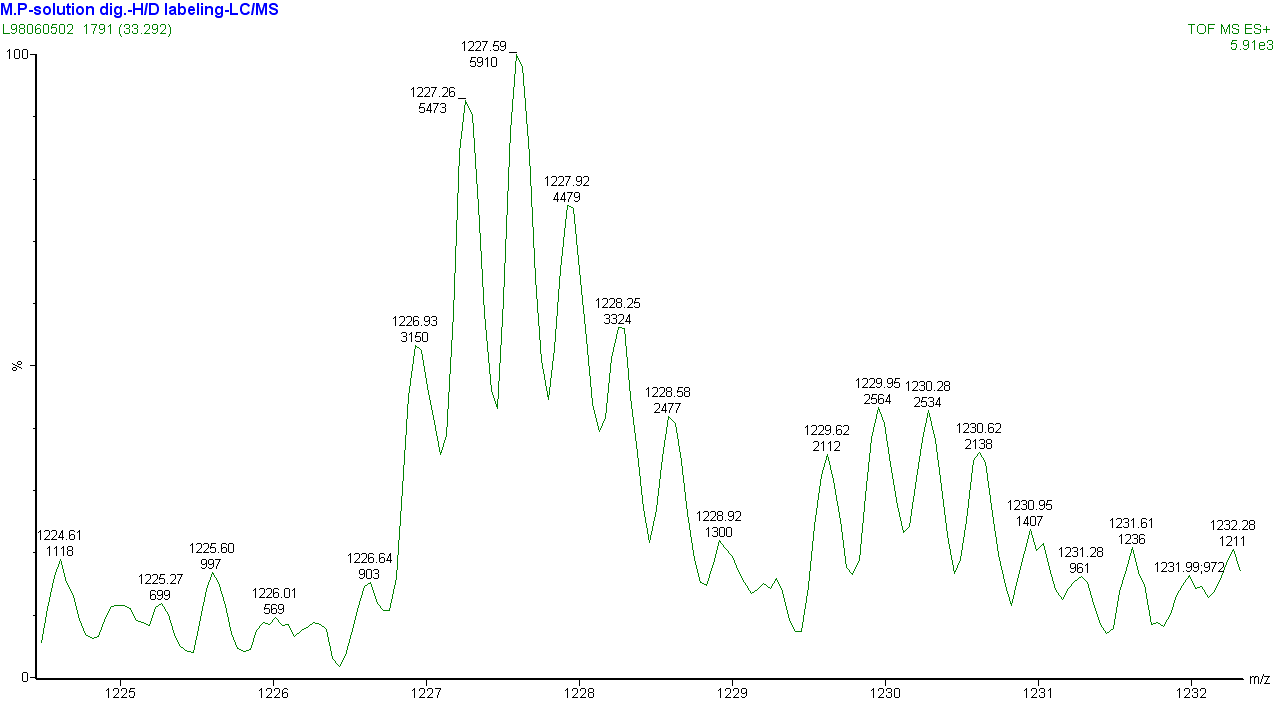


D/H ratio raw data of **Probable aquaporin PIP2**

***ALGSFGpSFGpSFR**


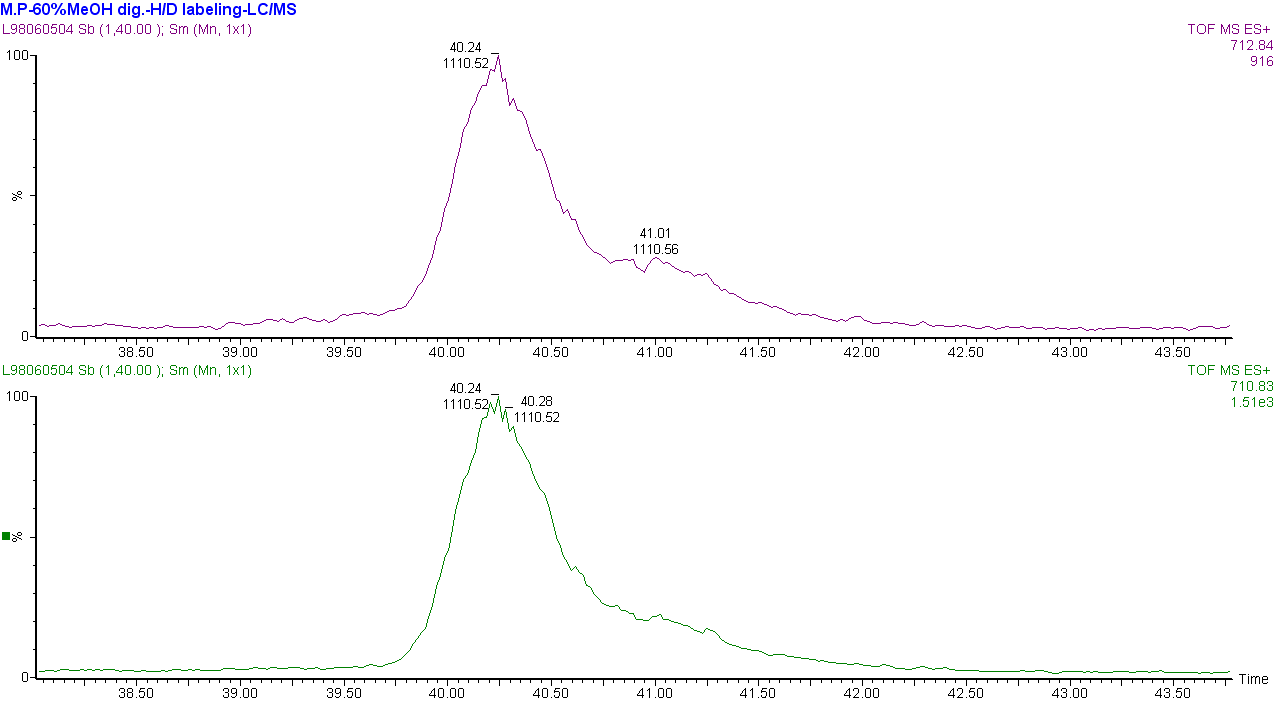


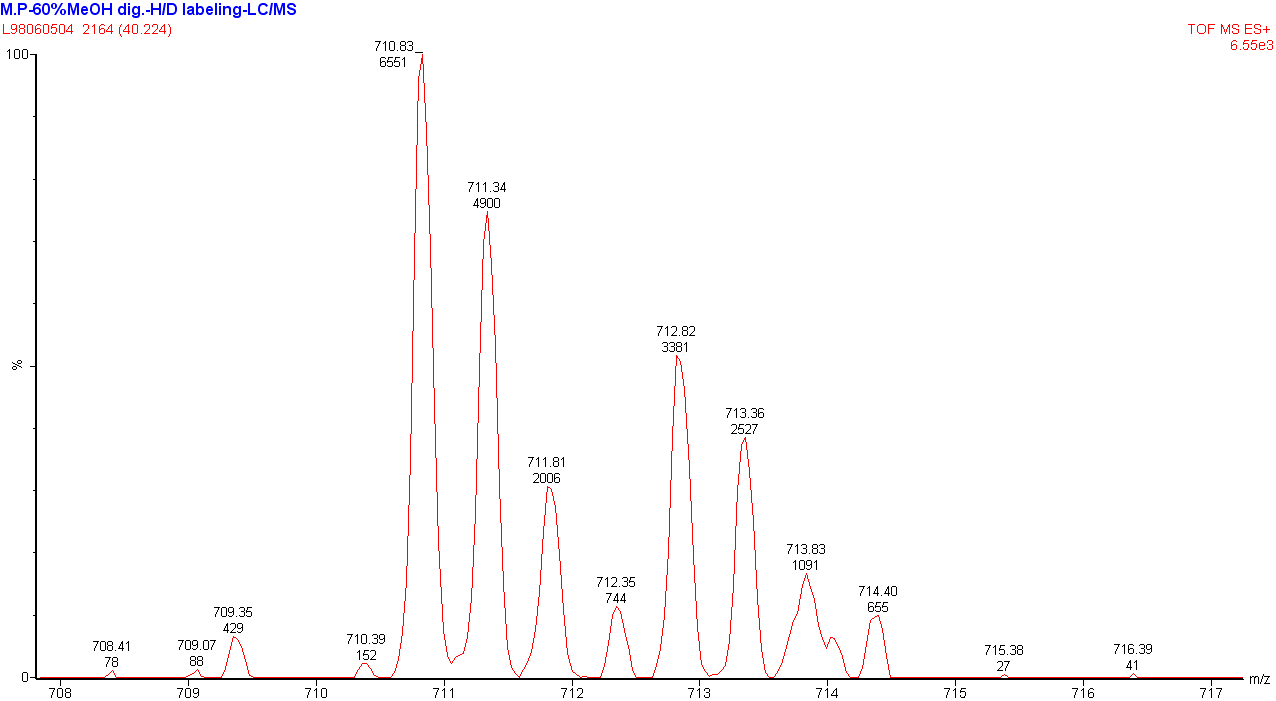


D/H ratio raw data of **Probable inactive receptor kinase**

***LIEEVSHSSGSPNPVpSD**

**
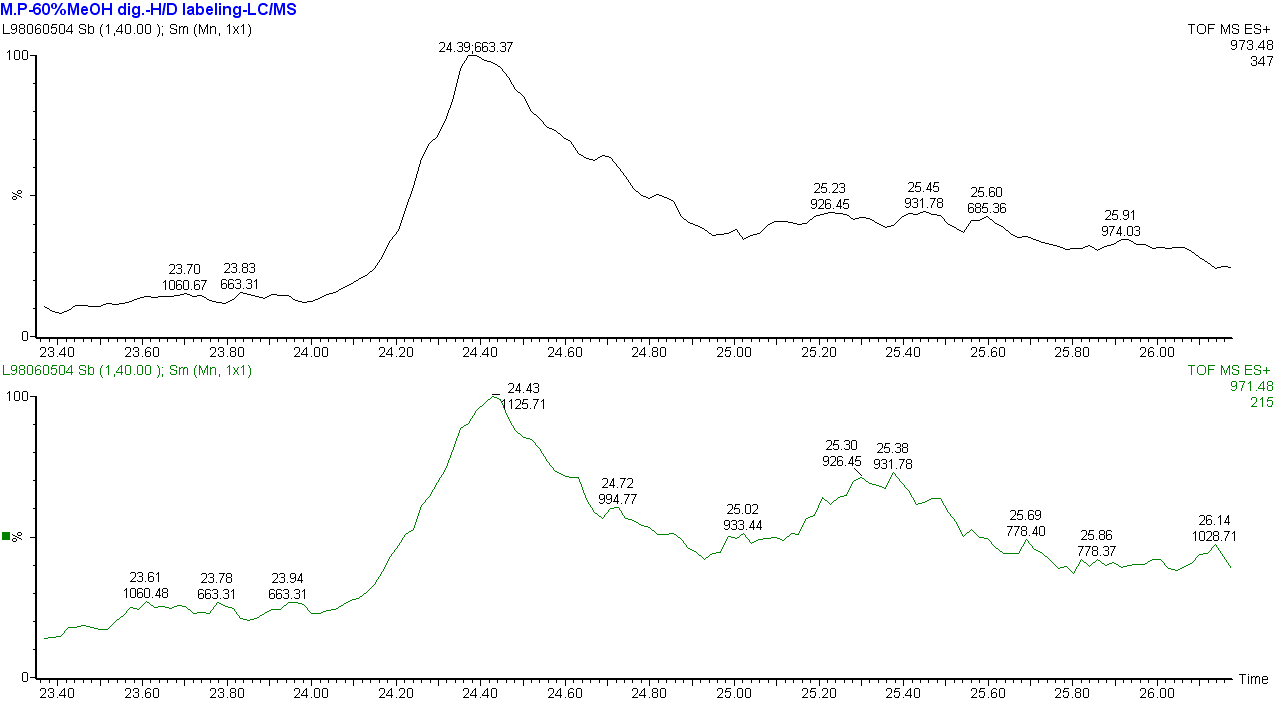
**

**
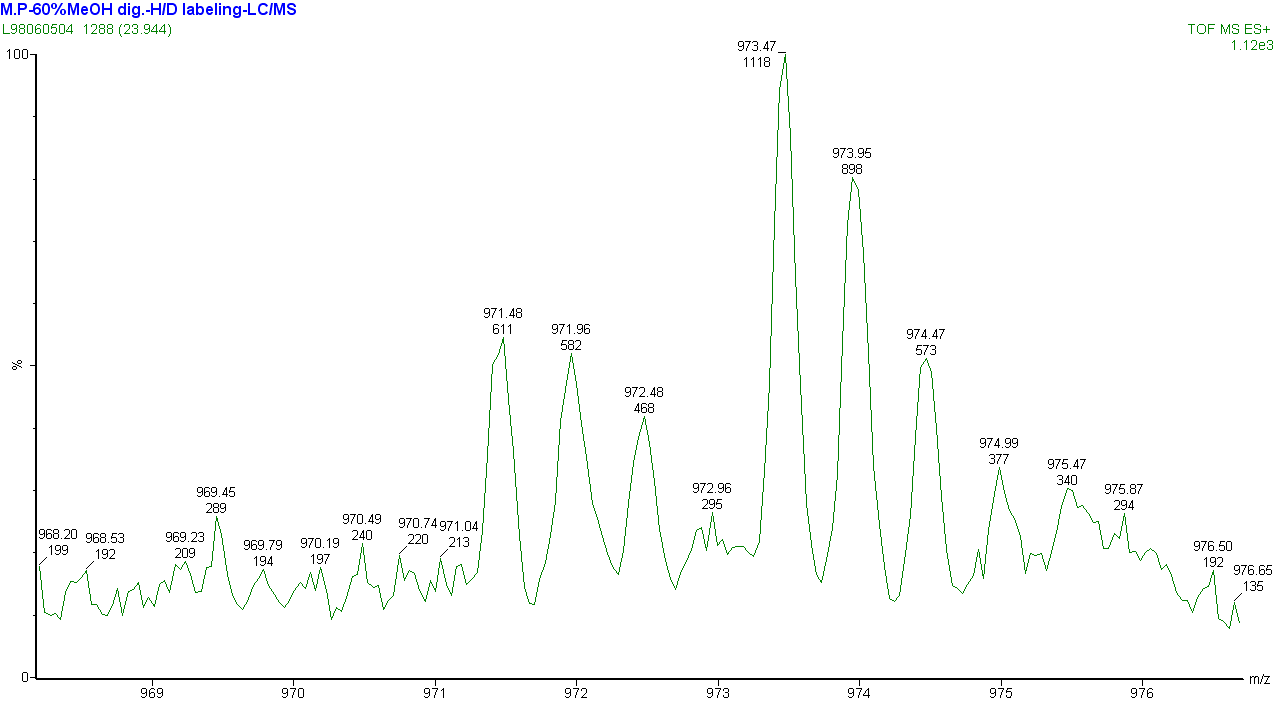
**

**Decoy search result**

**200mM NaCl**

**
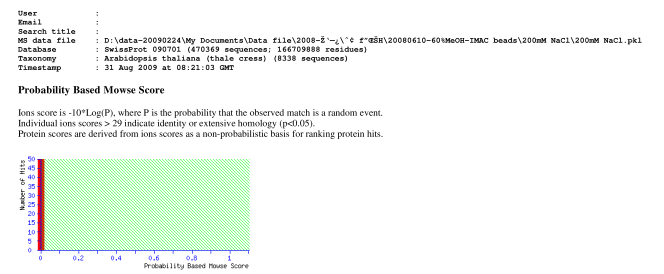
**

**Control**

**
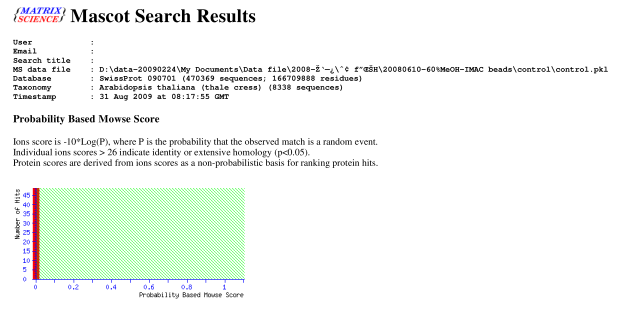
**
