# Supplementary material for: Functional phosphoproteomic profiling of phosphorylation sites in membrane fractions of salt-stressed Arabidopsis thaliana
Source: Proteome Sci. 2009 Nov 10;7:42. doi: 10.1186/1477-5956-7-42 (PMC2778640; doi:10.1186/1477-5956-7-42)

Supplemental Figure 1. MS/MS spectrum of SLGpSFRpSAANV (PIP22_ARATH)

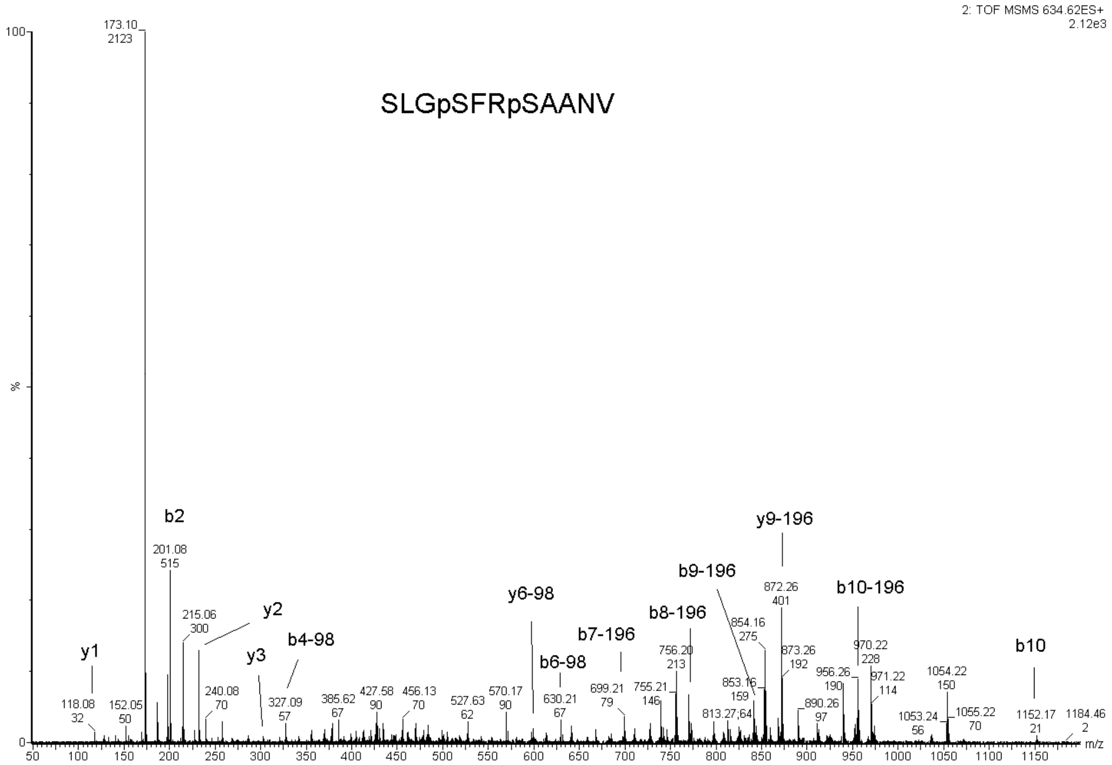

Supplemental Figure 2. MS/MS spectrum of QTTAEGSANPEPDQILpSPR (PUP18_ARATH)

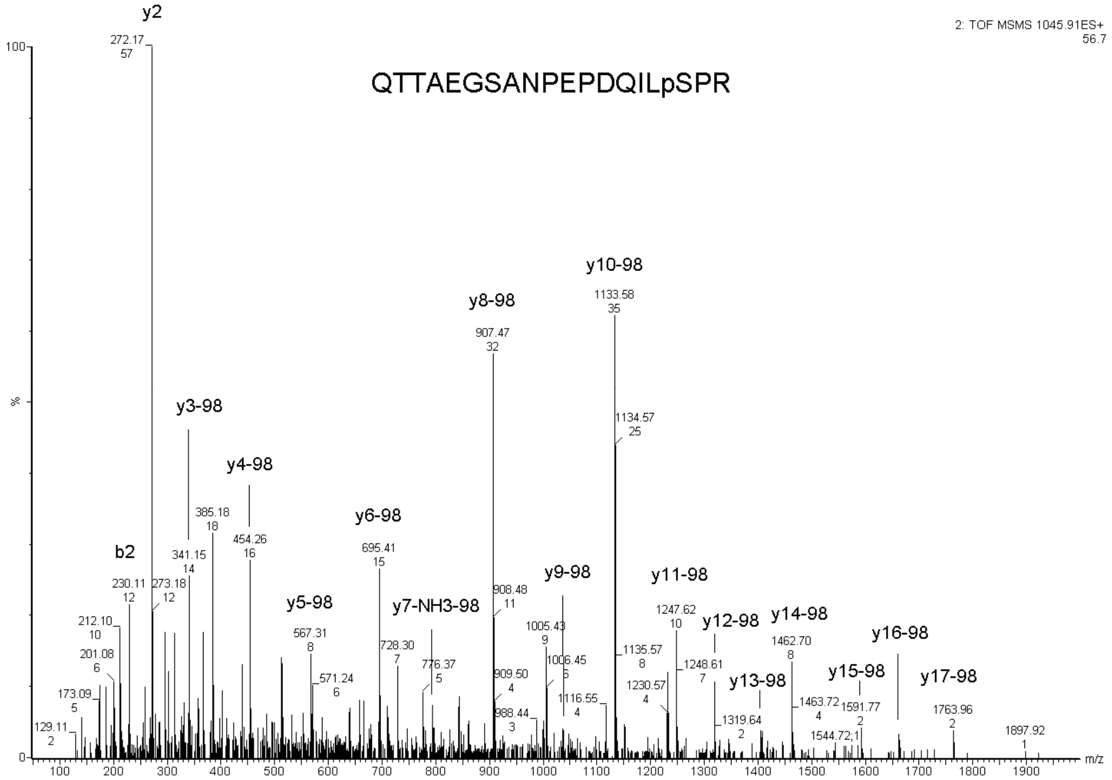

Supplemental Figure 3. MS/MS spectrum of LIEEVSHSSGSPNPVpSD and LIEEVSHSSGpSPNPVSD mixture (Y3288_ARATH)

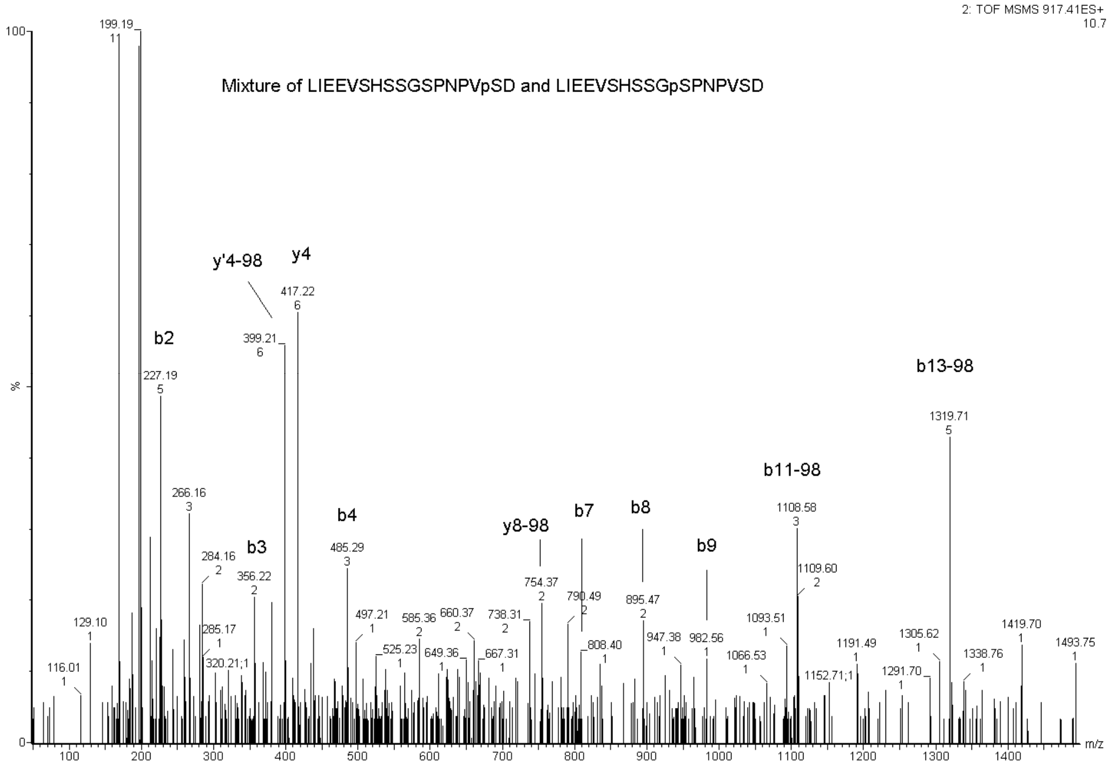

Supplemental Figure 4. MS/MS spectrum of ILQSEpSFKEEGYLASELQEAEK (PATL2_ARATH)

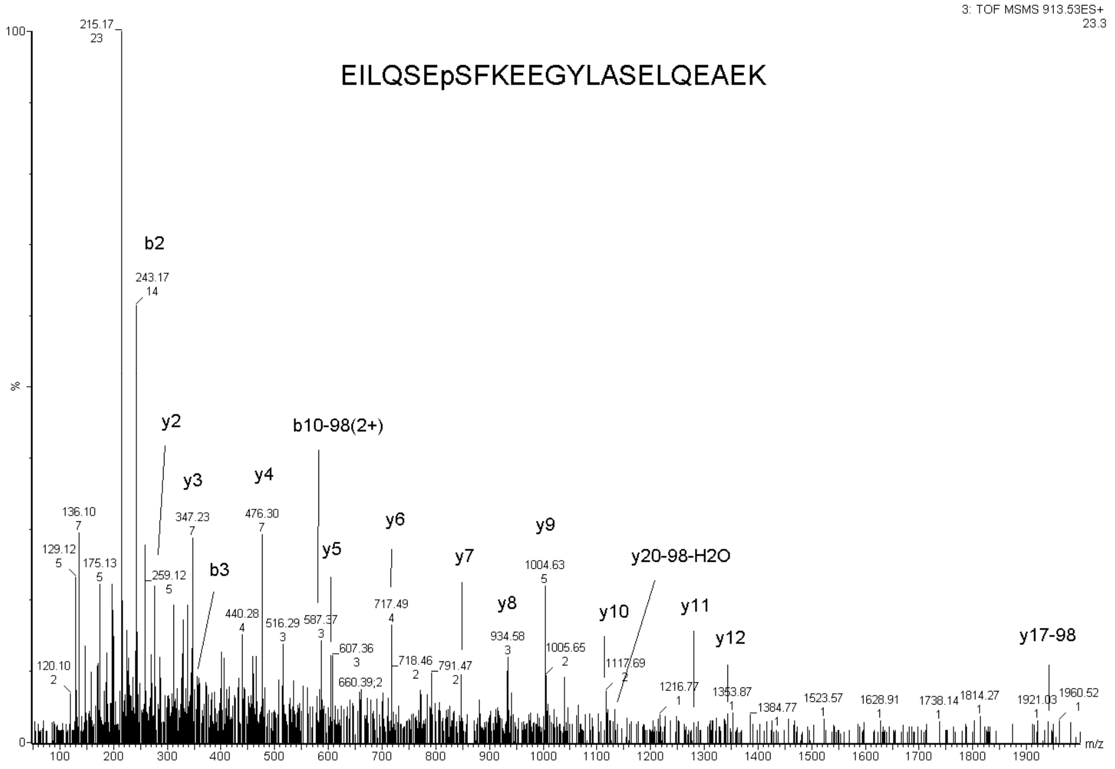

Supplemental Figure 5. MS/MS spectrum of GLDIDTAGHHYpTV (PMA1_ARATH)

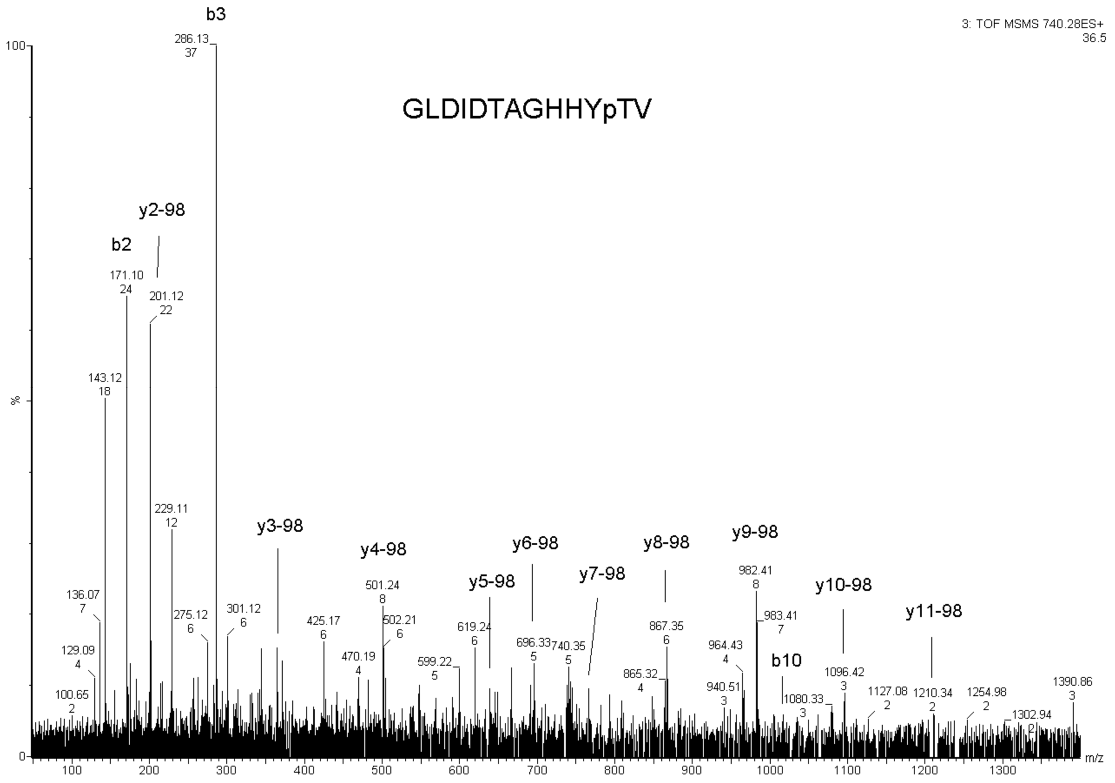

Supplemental Figure 6. MS/MS spectrum of DNDVPVpSYSGSGGPTK (Y1515_ARATH)

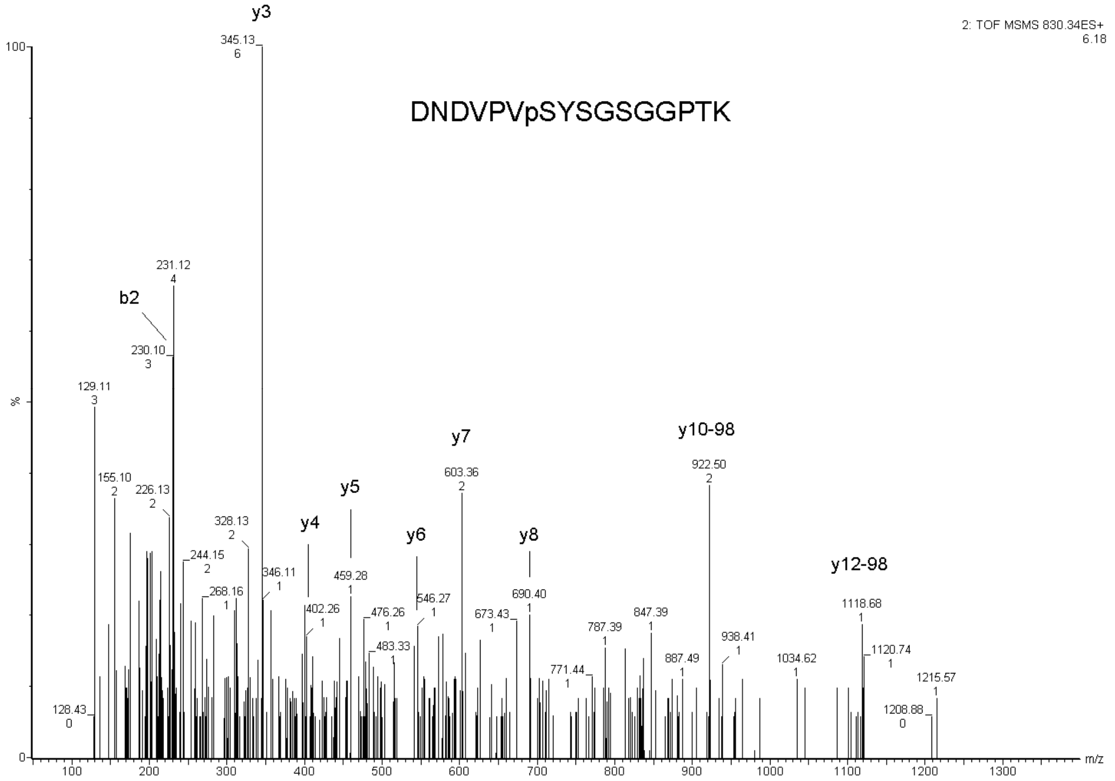

Supplement: Additional file 3 — MS/MS spectrum of six phosphopeptides. The file includes MS/MS spectrum of SLGpSFRpSAANV (PIP22_ARATH); MS/MS spectrum of QTTAEGSANPEPDQILpSPR (PUP18_ARATH); MS/MS spectrum of LIEEVSHSSGSPNPVpSD and LIEEVSHSSGpSPNPVSD mixture (Y3288_ARATH); MS/MS spectrum of EILQSEpSFKEEGYLASELQEAEK (PATL2_ARATH); MS/MS spectrum of GLDIDTAGHHYpTV (PMA1_ARATH); MS/MS spectrum of DNDVPVpSYSGSGGPTK (Y1515_ARATH). [file 1477-5956-7-42-S3.pdf]
